# Inflammation-targeted single-atom nanozymes drive microglial depolarization and inhibit ferroptosis via Sirt-6-xCT-GPX4 axis to attenuate early brain injury following subarachnoid hemorrhage

**DOI:** 10.1016/j.mtbio.2026.102829

**Published:** 2026-01-20

**Authors:** Boliang Liu, Chao Xiang, Xiaodan Zhang, Wei Guo, Haitao Wu, Fandi Hou, Yueyang Ba, Xiulei Zhang, Zhongcan Chen, Guang Feng, Yuan Dang, Yang Zhu, Jianjun Gu

**Affiliations:** a Department of Neurosurgery, People's Hospital of Zhengzhou University, Henan Provincial People's Hospital, Zhengzhou, Henan, People's Republic of China; b Department of Neurosurgery, Neurosurgery Research Institute, The First Affiliated Hospital, Fujian Medical University, Fujian Provincial Institutes of Brain Disorders and Brain Sciences, The First Affiliated Hospital, Fujian Medical University, Fuzhou, 35005, People's Republic of China; c Department of Neurosurgery, Henan Provincial People's Hospital Affiliated to Henan University, Zhengzhou, Henan, People's Republic of China; d Department of Clinical Medicine, Henan University, Kaifeng, Henan, People's Republic of China; e Department of Microbiome Laboratory, Henan Provincial People's Hospital, Zhengzhou University, Zhengzhou, Henan, People's Republic of China; f Innovation Center for Cancer Research, Clinical Oncology School of Fujian Medical University, Fujian Cancer Hospital, Fuzhou, People's Republic of China; g Fujian Key Laboratory of Advanced Technology for Cancer Screening and Early Diagnosis, Fujian Cancer Hospital, Fuzhou, People's Republic of China

**Keywords:** Subarachnoid hemorrhage, Single-atom nanozyme, Ferroptosis, Reactive oxygen species, Early brain injury

## Abstract

Early brain injury (EBI) has been identified as a key factor leading to the poor prognosis of patients with subarachnoid hemorrhage (SAH). At present, apart from surgical treatment, there is a lack of effective neuroprotective drugs. In this study, a biomimetic nanozyme V-MDL-800 was constructed by coordinating Vanadium Single-atom enzymes (V/SAE) and the allosteric activator MDL-800 of Sirt6, and encapsulated into NM@V-MDL-800 with neutropenia cell membrane (NM). By clearing ROS, the xCT/GPX4 pathway was activated, blocking the pathophysiological process of EBI after SAH can improve prognosis. NM@V-MDL-800 recruits through the blood-brain barrier (BBB) at the site of hemorrhagic injury by relying on the chemotactic property of neutrophils. Among them, the catalase-like, superoxide dismutase-like, and hydroxyl radical scavenging effects of V/SAE can eliminate excessive reactive oxygen species (ROS) within cells and inhibit oxidative stress; at the same time, as an allosteric activator of Sirt6, it activates the downstream xCT/GPX4 pathway, improving lipid metabolism abnormalities. Regulating the key core pathway of lipid peroxidation on ferroptosis promotes the polarization of microglia from the pro-inflammatory M1 form to the anti-inflammatory M2 morphology to inhibit the pathophysiological process of neuroinflammation in EBI. In addition, in vivo imaging of mice confirmed the targeted effect of NM@V-MDL-800 through the blood-brain barrier and recruited at the site of bleeding injury. The therapeutic effect of NM@V-MDL-800 on the SAH model has also been confirmed in vivo and in vitro experiments. This provides new ideas for SAH drug therapy regimens of SAH targeting microglial ferroptosis.

## Graphical abstract

**Figure:**
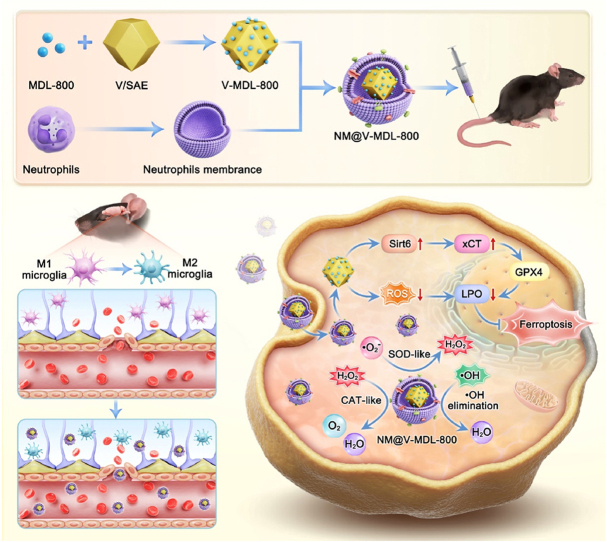

## 1 Introduction

Subarachnoid hemorrhage (SAH)is a severe hemorrhagic stroke, usually caused by the rupture of an aneurysm. It ranks third in incidence among all strokes and is one of the most devastating cerebrovascular diseases [1,2]. Early brain injury (EBI) is regarded as the leading cause of poor prognosis after SAH [3]. In recent years, although surgical methods for SAH have been continuously developed, due to the irreversible nature of nerve damage, the success of surgery often does not represent a good prognosis for patients [4,5]. Therefore, there is an urgent need to develop effective neuroprotective agents to improve EBI. After SAH, two pathophysiological processes, oxidative stress caused by intracellular reactive oxygen species (ROS) accumulating excessively within cells and lipid metabolism disorders mediated by the xCT/GPX4 pathway caused lipid peroxidation, which is one of the main pathophysiological mechanisms of the ferroptosis pathway in EBI [[6], [7], [8], [9], [10]]. In addition, microglia are classified into resting state (M0), pro-inflammatory state (M1), and anti-inflammatory state (M2) [[11], [12], [13], [14]]. Current research indicates that ferroptosis is a key driver in promoting microglial polarization to the M1 state [15]. Therefore, inhibiting ferroptosis and regulating the polarization of microglia from the pro-inflammatory M1 state to the anti-inflammatory M2 state is a promising therapeutic strategy to improve the prognosis of SAH [[16], [17], [18]]. However, because of the blood-brain barrier (BBB), most neuroprotective agents cannot be targeted to reach the damaged areas of the nervous system. Astrocytes, pericytes, and extracellular matrix (ECM) components provide both structural and functional support to the BBB, essential for maintaining homeostatic balance in the brain; however, at the same time, the presence of the BBB significantly limits the delivery of therapeutic agents into the brain [19,20]. Most systemically administered drugs, especially macromolecules and hydrophilic compounds, cannot pass through the BBB at clinically effective concentrations. Combined with the susceptibility to inactivation and short half-life of most natural invertases, these factors collectively contribute to the inefficiency of existing drugs [[20], [21], [22]]. Therefore, there is an urgent need to develop a drug that can target the bleeding injury site, inhibit ferroptosis, and promote the polarization of microglia to the M2 state.

Single-Atom Vanadium Enzymes(V/SAE) are a type of artificial mimicase that penetrates vanadium (V) atoms in the form of single atoms [23]. Its atomic-level dispersion ensures the maximization of atomic utilization efficiency of V/SAE, and it also has excellent catalase-like effects, superoxide dismutase-like effects, and hydroxyl radical scavenging [[24], [25], [26]]. Previously, studies have reported the application prospects of vanadium enzymes in anti-tumor, anti-inflammatory, and improving neurodegenerative diseases [26,27]. Sirtuin6 (Sirt6) is an NAD-dependent histone deacetylase that is involved in a variety of biological pathways. MDL-800 is an allosteric activator of Sirt6 that can exert neuroprotective functions upon activation [[28], [29], [30]]. Neutrophils are the first cells to be recruited into the brain after SAH. They naturally recruit to the site of the inflammatory response through chemotaxis, so the neutrophil membrane can give nanoparticles the ability to through BBB barrier target the site of neuroinflammation, which is very suitable for the requirement of inhibiting neuroinflammatory damage after SAH [31,32]. The nanozyme drug delivery system based on neutrophil membrane can not only use the fat solubility of the cell membrane to pass through the BBB, but also use the chemotaxis of inflammatory response cells to target drug delivery, and then maximize the effect of nanozymes, indicating that the neutrophil-based nanodrug delivery system may be a promising new strategy for drug therapy after SAH [33,34].

We used hydrophobic interactions to load MDL-800 onto V/SAE, and then wrapped it with neutrophil membranes to construct a biomimetic nanozyme NM@V-MDL-800, which gives it the ability to penetrate the BBB and target delivery to the site of neuroinflammatory injury after hemorrhage, and then clear ROS and activate Sirt6 and its downstream pathways to provide neuroprotective effects [[35], [36], [37], [38]]. V/SAE has efficient catalase-like, superoxide dismutase-like, and hydroxyl radical scavenging, which can quickly remove ROS and inhibit oxidative stress [39,40]. With Sirt6 activation, the effect of System Xc-can be enhanced through the xCT/GPX4 pathway, transporting excess glutamate (Glu) out of the cell and cystine (Cys) into the cell, promoting glutathione (GSH) synthesis, as a core antioxidant within cells, GSH neutralizes reactive oxygen species, and maintains redox homeostasis, regulates intracellular lipid metabolism to inhibits ferroptosis and promotes the conversion of microglia from M1 to M2, and reduces neuroinflammation [6,[41], [42], [43]]. In addition, in vivo imaging of mice showed that anthocyanin-5 (Cy5)-labeled NM@V-MDL-800 had excellent BBB penetration and targeted aggregation at the site of bleeding injury in the SAH model. In vitro experiments have shown that NM@V-MDL-800 can activate Sirt6, protect microglia, and improve cell survival. In vivo experiments have shown that NM@V-MDL-800 can improve the survival rate after SAH and improve cognitive and memory function. This study not only provides validation for the development of NM-encapsulated biomimetic nanozyme drugs but also reveals their role in improving EBI, providing a new perspective for clinical drug treatment of SAH.

## 2 Results and discussion

### 2.1 Synthesis and characterization of V-MDL-800 and NM@V-MDL-800

**Scheme 1:**
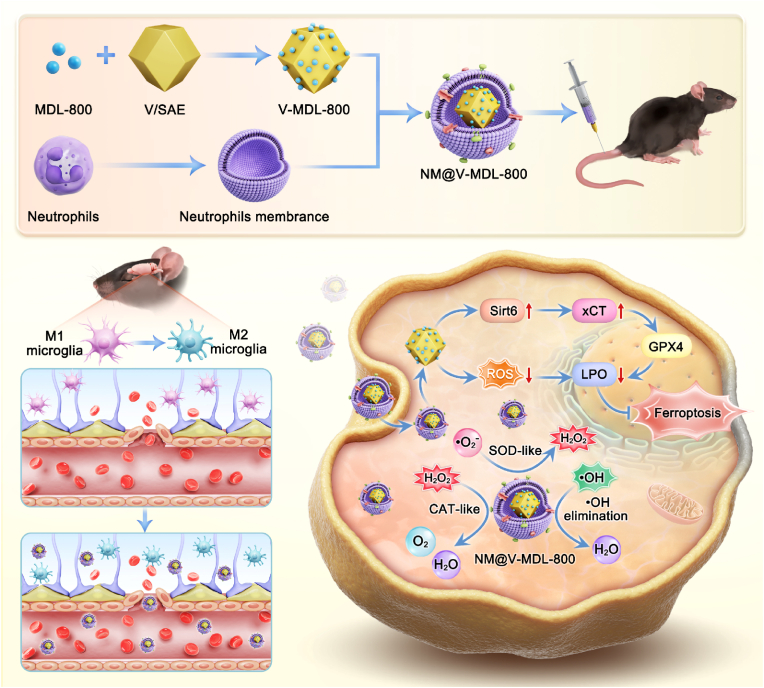
In the SAH model, intravenous injection through the tail vein NM@V-MDL-800 can alleviate oxidative stress, activate Sirt6 and its downstream pathways, inhibit iron hoisting, regulate microglial polarization, and improve prognosis.

**Fig. 1:**
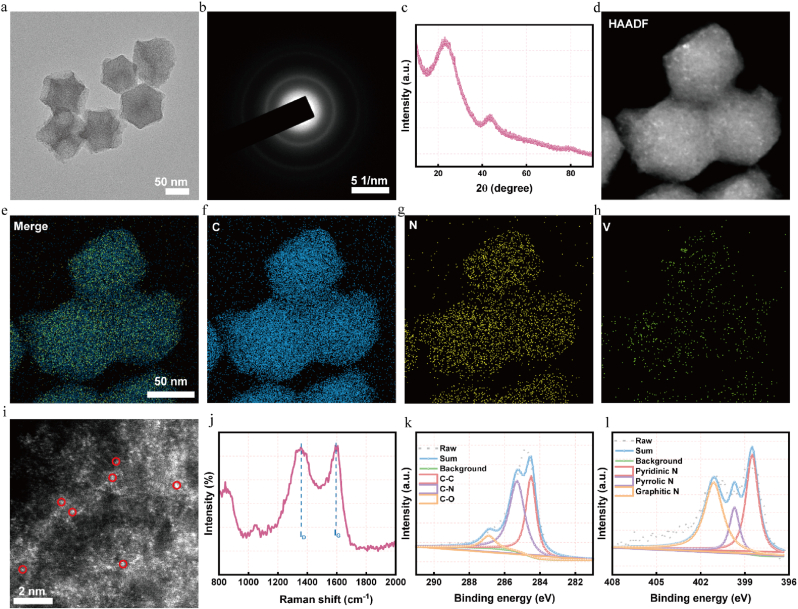
**Comprehensive characterization of NM@V-MDL-800.** (a) TEM image of V/SAE. (b) SAED image of V/SAE. (c) XRD pattern of V/SAE. (d–h) EDX mapping images of C, N, V atoms and their merge in V/SAE. (i) Magnified HAADF-STEM image of V/SAN showing single-atomic V as bright dots. (j) Raman spectrum of V/SAE. (k) High-resolution C 1s XPS spectrum. (l) High-resolution N 1s XPS spectrum.

The complex process of synthesizing V/SAE is depicted in detail in Scheme 1. First, V/SAE is prepared by pyrolysis V@ZIF-8 precursor. Subsequently, Vanadium acetylacetone (V(acac)_3_) is encapsulated in situ in a ZIF-8 body cage, effectively integrating vanadium precursors into a metal-organic framework. Transmission electron microscopy (TEM) images show that the V@ZIF-8 precursor still maintains the shape of a rhomboid dodecahedron (Fig. S1). After annealing the V@ZIF-8 derivative, V/SAE was successfully obtained. High-resolution TEM image displays that V/SAE exhibits a uniform dodecahedral shape with a diameter of approximately 90 nm. After 950° treatment, the surface of V/SAE becomes rough and porous (Fig. 1a). These selective region electron diffraction (SAED) images corresponding to V/SAE show two diffused diffraction rings, indicating that the N-C nano-framework is amorphous (Fig. 1b). The X-ray diffraction (XRD) spectra show that neither the metal V nor the V oxide has crystalline peaks, indicating that there is no obvious V aggregation, which supports the successful preparation of single-atom V nanozyme (Fig. 1c). Energy dispersive X-ray spectroscopy (EDS) imaging revealed a uniform distribution of V, C, and N atoms within V/SAE (Fig. 1d–h). The corrected aberration of the atomic resolution high-angle annular dark-field scanning transmission electron microscope (HAADF-STEM) shows bright spots, highlighted by red circles, representing the V of atomic distribution (Fig. 1i). Furthermore, Raman analysis indicates that the crystallinity of the nitrogen-carbon layer after pyrolysis is poor and there are numerous defects, which is conducive to stabilizing the vanadium atoms dispersed by atoms (Fig. 1j). The iron loading efficiency of V/SAE was determined to be 1.17 by inductively coupled plasma mass spectrometry (ICP-MS). V/SAE-MD-800 was prepared by encapsulating MD-800 in V/SAE through hydrophobicity. These results confirmed the successful synthesis of V/SAE-MDL-800.

**Fig. 2:**
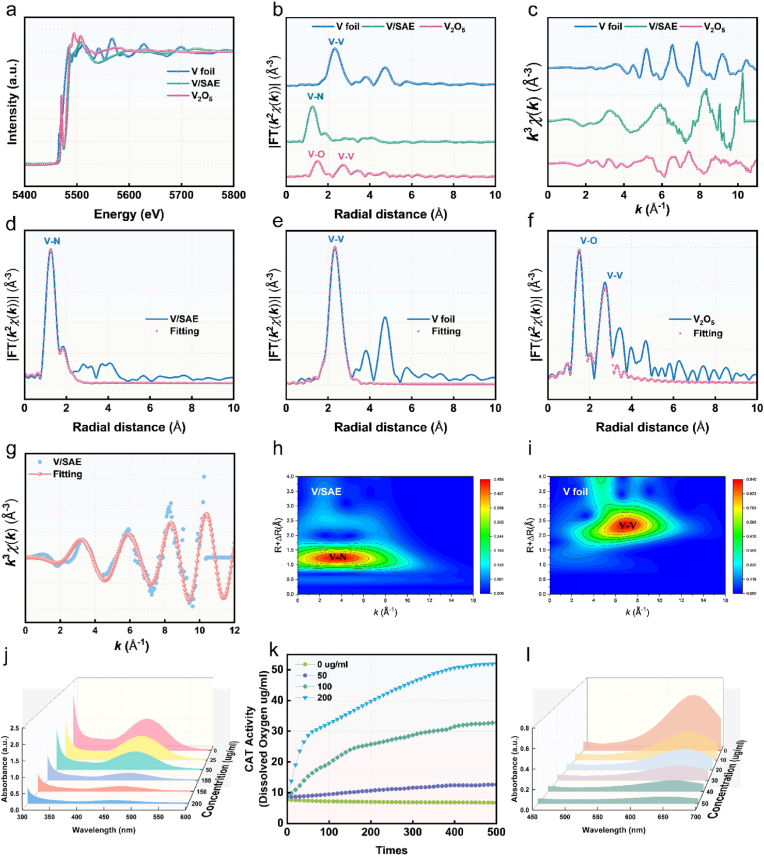
**Atomic structural characterization and enzymatic performance of NM@V-MDL-800.** (a) Normalized V K-edge XANES spectra of V foil, V/SAE, and V_2_O_5_. (b) The Fourier transform EXAFS of the V K-edge of V foil, V/SAE, and V_2_O_5_. (c) EXAFS curves of V foil, V/SAE, and V_2_O_5_ at the K space. (d) EXAFS fitting curve of V/SAE and (e) V foil and(f) V_2_O_5_ at the R space. (g) EXAFS fitting curves of V/SAE in the k space. (h)Wavelet transformation of V K-edge EXAFS of V/SAE and (i) V foil. (j) Evaluation of SOD-like activity of NM@V-MDL-800. Superoxide scavenging capacity of different catalyst concentrations (0–200 μg/mL). (k) Evaluation of the CAT-like activity of NM@V-MDL-800. Time-course curves of dissolved oxygen concentration during H_2_O_2_ decomposition by different concentrations of catalyst (0–200 μg/mL).(l) Evaluation of HORAC activity of NM@V-MDL-800 Fenton reaction mediated by catalyst (0–50 μg/mL), absorption spectra in the wavelength range of 450–700 nm demonstrating concentration-dependent hydroxyl radical decomposition.

The valence states of carbon, vanadium, and nitrogen atoms in V/SAE were evaluated using X-ray photoelectron spectroscopy (XPS). As shown in Fig. 1k, the C 1s spectra of V/SAE show mainly characteristics corresponding to sp2 hybridized graphitic carbon (Fig. 1k). The N1s spectra reveal three different nitrogen species: graphitic nitrogen, pyridine nitrogen, and pyrrolines. It is worth noting that a large number of pyridine nitrogen species have been observed. This high content of pyridine nitrogen is significant as it provides a coordination site for the fixation of single-atom vanadium (Fig. 1l), a feature that is crucial for enhancing the catalytic activity of V/SAE. To determine the coordination number of vanadium, synchrotron X-ray absorption near-end structure (XANES) and extended X-ray absorption fine structure (EXAFS) analyses were conducted. The V K-edge XANES spectrum shows the energy absorption threshold (Fig. 2a), confirming the V^δ+^ oxidation state, which is consistent with the XPS results. Furthermore, the Fourier transform of the EXAFS data in the R space has a significant peak at 1.48a, corresponding to the V-N key. It is worth noting that no peak was detected at 2.35 Å^−1^, which may indicate the existence of V-V bonding. These results clearly confirm the presence of atomically dispersed vanadium active sites in V/SAE (Fig. 2b–f). The EXAFS fitting spectra of the vanadium k-edge show a fine structure, indicating that the vanadium atoms in V/SAE form coordination with four nitrogen atoms (Fig. 2g–S2-S3, Table S1). Furthermore, V/SAE exhibited a wavelet transform (WT) signal at 3.9 Å^−1^, corresponding to the vanadium-nitrogen bond, without the wavelet transform intensity associated with the vanadium-vanadium bond (Fig. 2h–i and S4). To sum up, these results prove the successful construction of V/SAE.

### 2.2 NM@V-MDL-800 showed remarkable enzyme-mimicking activities

The SOD-like activity of NM@V-MDL-800 was assessed using a WST-8 assay kit. Results showed a dose-dependent increase in the inhibition rate of superoxide anion radicals (·O_2_^−^)(Fig. 2j), confirming its concentration-dependent ·O_2_^−^ scavenging capability. Real-time monitoring of oxygen release dynamics during H_2_O_2_ decomposition was performed via dissolved oxygen measurement. Higher NM@V-MDL-800 concentrations resulted in a significant increase in dissolved oxygen levels, indicating strong CAT-like activity(Fig. 2k). NM@V-MDL-800 showed typical HORAC activity via the potassium ferricyanide colorimetric method. Its capacity to degrade peroxides via the Fenton reaction was strongly correlated with its concentration(Fig. 2l). As shown in Fig. S5, DPPH radical scavenging experiments indicated that the scavenging efficiency of DPPH radicals significantly increased with higher NM@V-MDL-800 concentrations, highlighting its potent free radical elimination properties.

### 2.3 In vitro NM@V-MDL-800 restores cell viability in a hemin-induced model of microglial SAH injury

**Fig. 3:**
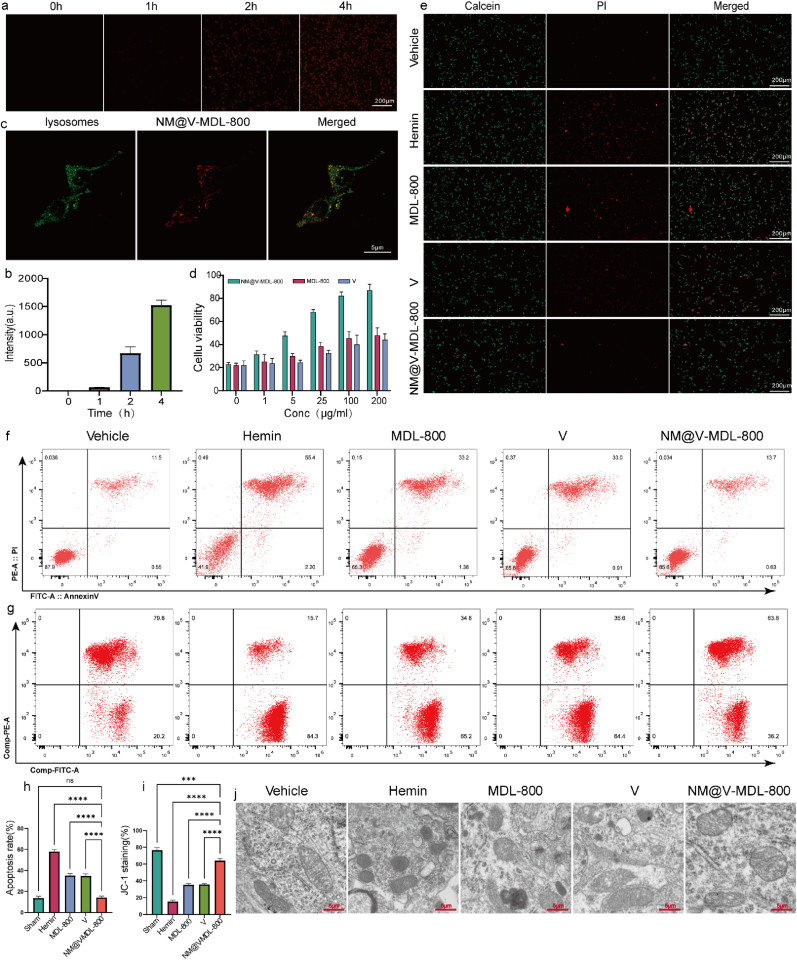
**The in vitro protective effects of NM@V-MDL-800.** (a) CLSM images of NM@V-MDL-800 in BV-2 cells. (b)Quantitative analysis of fluorescence intensity. (c)CLSM images of colocalization between LysoTracker (green fluorescence) and Cy5.5-labeled NM@V-MDL-800 (red fluorescence) in BV-2 cells after 4 h of incubation. (d) Cell viability after 24 h of treatment with different concentrations of NM@V-MDL-800, V, and MDL-800 in Hemin-induced BV-2 cells. (e) CLSM images of Calcein-AM/PI colocalization staining after 24 h of treatment in each group. (f–i) Cell apoptosis and JC-1 staining shows mitochondrial membrane potential was evaluated using the flow cytometry. (j) Transmission electron microscopy (TEM) images of BV2 cell^'^s mitochondria. (For interpretation of the references to color in this figure legend, the reader is referred to the Web version of this article.)

We selected BV-2 cells and used heme as the inducer to construct an in vitro SAH model of microglia [10]. At the same time, a variety of methods were used to detect the in vitro protective effect of NM@V-MDL-800. First, we used Confocal Laser Scanning Microscopy (CLSM) to observe the uptake of NM@V-MDL-800 by Hemin-treated BV-2 cells at different time points. The results showed that the fluorescence intensity of the red-fluorescent NM@V-MDL-800 gradually increased over time, indicating its ability to enter cells and exert pharmacological effects(Fig. 3a–b).Colocalization experiments showed that Cy5.5-labeled NM@V-MDL-800 could effectively escape from lysosomes to the cytoplasm, thereby exerting its function of ROS scavenging (Fig. 3c).The CCK8 experiment showed that the survival rate of BV-2 cells treated with NM@V-MDL-800 was significantly higher than that of cells treated with free vanadium single-atom enzymes (V) or free MDL-800 alone. This trend is becoming more pronounced as the dosing concentration gradually increases from 0 μg/ml to 200 μg/ml, and the cell survival rate increases from about 20 % to about 90 % (Fig. 3d). This indicates that NM@V-MDL-800 has a significant effect on improving cell survival, and the efficacy is concentration-dependent. In addition the in vitro protective effect of NM@V-MDL-800 was detected by Calcein-AM/Propidium iodide (PI) co-staining, and the results showed that it significantly improved the cell survival rate, and the effect was better than that of the free V group and the MDL-800 group, which was consistent with the results of the CCK8 experiment (Fig. 3e).Furthermore, according to flow cytometry results, apoptosis in BV-2 cells was significantly elevated and mitochondrial membrane potential was significantly decreased after hemin induction, and treated with NM@V-MDL-800 significantly reduced the apoptosis rate and restore mitochondrial membrane potential compared with the MDL-800 and V group(Fig. 3f–i). Then TEM results shown that hemin induction decreased or absent mitochondrial crest, ruptured mitochondrial membranes and mitochondrial shrinkage and NM@V-MDL-800 reduced this injury (Fig. 3j). In conclusion, these results confirm that NM@V-MDL-800 can significantly increase the survival rate of BV-2 cells in the SAH model, indicating that it can effectively inhibit neuronal cell death in vitro.

### 2.4 In vitro, the role of NM@V-MDL-800 on microglial SAH models

**Fig. 4:**
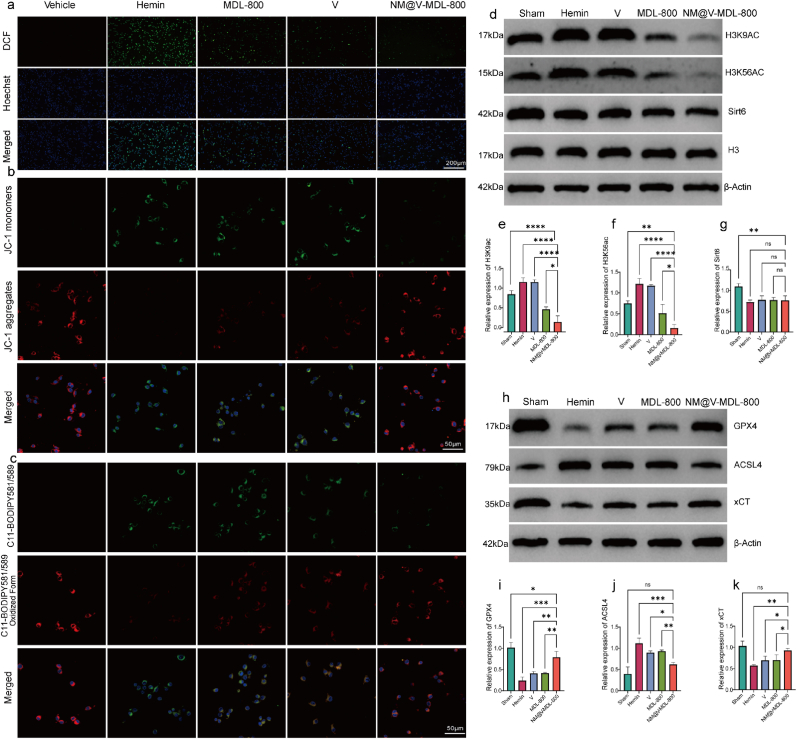
**The effect and mechanism of NM@V-MDL-800 on microglial SAH models.** (a) CLSM images of DCFH-DA staining of BV-2 cells after treatment with different agents and incubation for 4 h. (b) CLSM images of JC-1 staining of BV-2 cells after treatment with different agents and incubation for 4 h. (c) CLSM images of C11-BODIPY581/589 staining of BV-2 cells after treatment with different agents and incubation for 24 h. (d) Western blot analysis of H3K9ac, H3K56ac, and Sirt6 in BV-2 cells treated with different agents and incubated for 24 h. (e–g) Relative expression levels. (h) Western blot analysis of GPX4, ACSL4, and xCT. (i–k) Relative expression levels.

Based on the cytoprotective properties exhibited by NM@V-MDL-800 in vitro experiments, this study delves into the critical role of oxidative stress and abnormal lipid metabolism in inflammatory damage during SAH. Firstly, the fluorescent indicator (2′,7′-dichlorofluorescin diacetate or DCFH-DA) of microglia showed that ROS levels were significantly increased in microglia in the SAH model, while the ROS levels were significantly reduced after treatment with NM@V-MDL-800 (Fig. 4a and S6). This confirms the significant benefits of NM@V-MDL-800 in reducing ROS levels. In addition, NM@V-MDL-800 effectively mitigates of Mitochondrial Membrane Potential (MMP) polarization and mitochondrial dysfunction due to excessive ROS production in BV-2 cells. Fluorescence intensity changes are monitored by the fluorescent probe JC-1. It was confirmed that the mitochondrial membrane potential was restored (Fig. 4b). In terms of improving lipid metabolism abnormalities, co-staining of microglia using the fat-soluble fluorescent probe C11-BODIPY^581/589^ and its oxidized form observed a significant increase in oxidized lipid unsaturated bonds in SAH model microglia, while NM@V-MDL-800 inhibited ROS attack on lipid unsaturated bonds and improved lipid metabolism abnormalities in microglia (Fig. 4c). To evaluate the effect of NM@V-MDL-800 on ferroptosis-associated lipid peroxidation, we measured the level of malondialdehyde (MDA), a terminal product of lipid peroxidation. The results showed that after SAH, the MDA level of BV-2 cells increased significantly, and the addition of NM@MDL-800 significantly reversed this process (Fig. S7). To verify the activation and action ability of NM@V-MDL-800 on Sirt6, we used Western blot (WB) to detect the expression levels of Sirt6 and acetylated proteins H3K9ac and H3K56ac. The expression level of Sirt6 decreased after SAH, while the expression levels of H3K9ac and H3K56ac increased. After treatment with MDL-800 and NM@V-MDL-800, the expression of acetylated level proteins H3K9ac and H3K56ac decreased, and the activation effect was more significant in the NM@V-MDL-800 treatment group, indicating that NM@V-MDL-800 can effectively activate Sirt6 (Fig. 4d–g). Based on the above experimental results, it is speculated that the therapeutic effect of NM@V-MDL-800 on the SAH microglial model may be related to ferroptosis, so the expression of proteins involved in ferroptosis is further detected to elucidate the mechanism by which NM@V-MDL-800 inhibits ferroptosis. Expression levels of xCT, ACSL4, and GPX4 were assessed by Western blot (WB). The results showed that the NM@V-MDL-800 group inhibited ACSL4 expression while increasing xCT and GPX4 expression, which was consistent with the expected results. And the therapeutic effect was superior to that of the free MDL-800 group and the free V/SAE group (Fig. 4h–k). This indicates that NM@V-MDL-800 can effectively regulate the expression levels of xCT, ACSL4, and GPX4, thereby inhibiting ferroptosis.

**Fig. 5:**
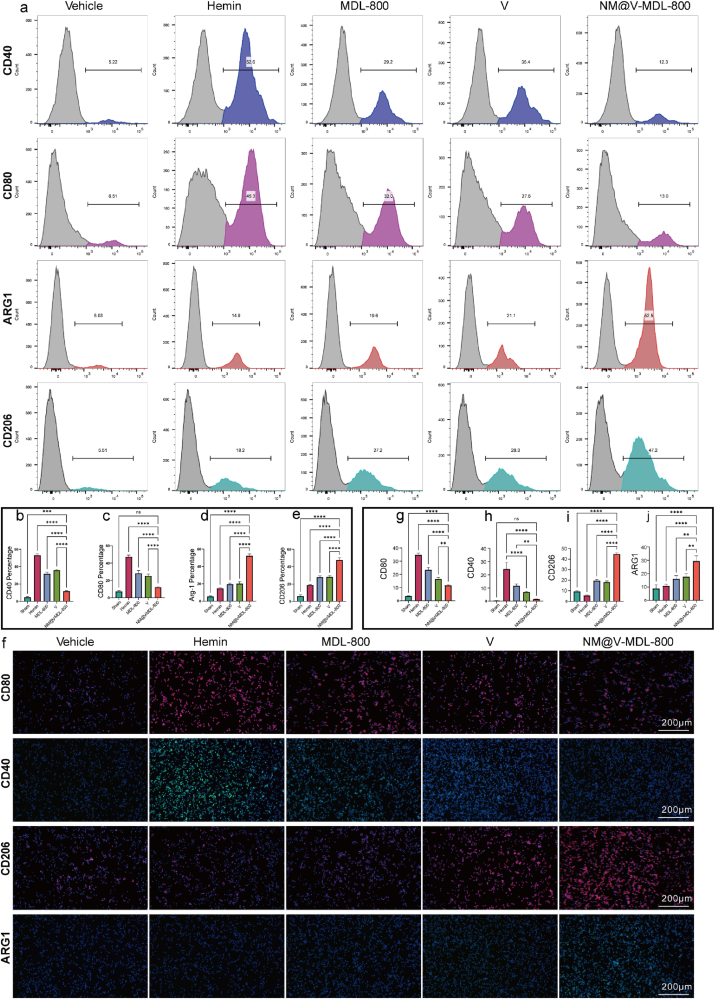
**Effect of NM@V-MDL-800 on M1/M2 polarization of microglia.** (a–e) Flow cytometry analysis and corresponding quantification markers for CD80, CD40, CD206, and ARG-1 after treatment with different agents and incubation for 24 h in BV-2 cells. (f–j) CLSM images of fluorescence and average fluorescence intensity corresponding to quantification markers for CD80, CD40, CD206, and ARG1 in BV-2 cells ∗∗∗P < 0.001. ∗∗P < 0.01. ∗P < 0.05.

Microglia can activate into two phenotypes, pro-inflammatory M1-like and anti-inflammatory M2-like, after SAH occurs. Ferroptosis inhibitors can interfere with microglial polarization and promote M1-like to M2-like transformation. CD40 and CD80 were selected as M1-like microglia polarization markers, and Arg-1 and CD206 were selected as M2-like microglia polarization markers and the potential of NM@V-MDL-800 in the regulation of microglial polarization was investigated using flow cytometry and. After treatment with NM@V-MDL-800, the expression of CD40 and CD80 in BV-2 cells was significantly down-regulated, while the expression of Arg-1 and CD206 was significantly increased, indicating that SAH model BV-2 cells may be polarized to M2-like phenotype under NM@V-MDL-800 intervention, and this process is closely related to its inhibition of ferroptosis (Fig. 5a–e). The results of the CLSM image were consistent with flow cytometry (Fig. 5f–j and S8-11). Subsequently, in order to verify the relationship between ferroptosis and microglia polarization, we added the ferroptosis inducer RSL3 on the basis of injecting NM@V-MDL-800 after SAH. We performed RT-qPCR experiments to detect the mRNA expression of M1/M2 microglia-related markers. The results showed that RSL3 reversed the anti-inflammatory effect of NM@V-MDL-800 in promoting M2 polarization of microglia (Fig. S12). In vitro experimental results show that NM@V-MDL-800 shows a comprehensive cytoprotective effect on SAH model microglia by reducing oxidative stress and improving lipid metabolism disorders to inhibiting ferroptosis, and regulating microglia polarization, providing a promising new method for SAH treatment.

### 2.5 Biosafety and biodistribution after intravenous injection of NM@V-MDL-800 in the tail of mice

**Fig. 6:**
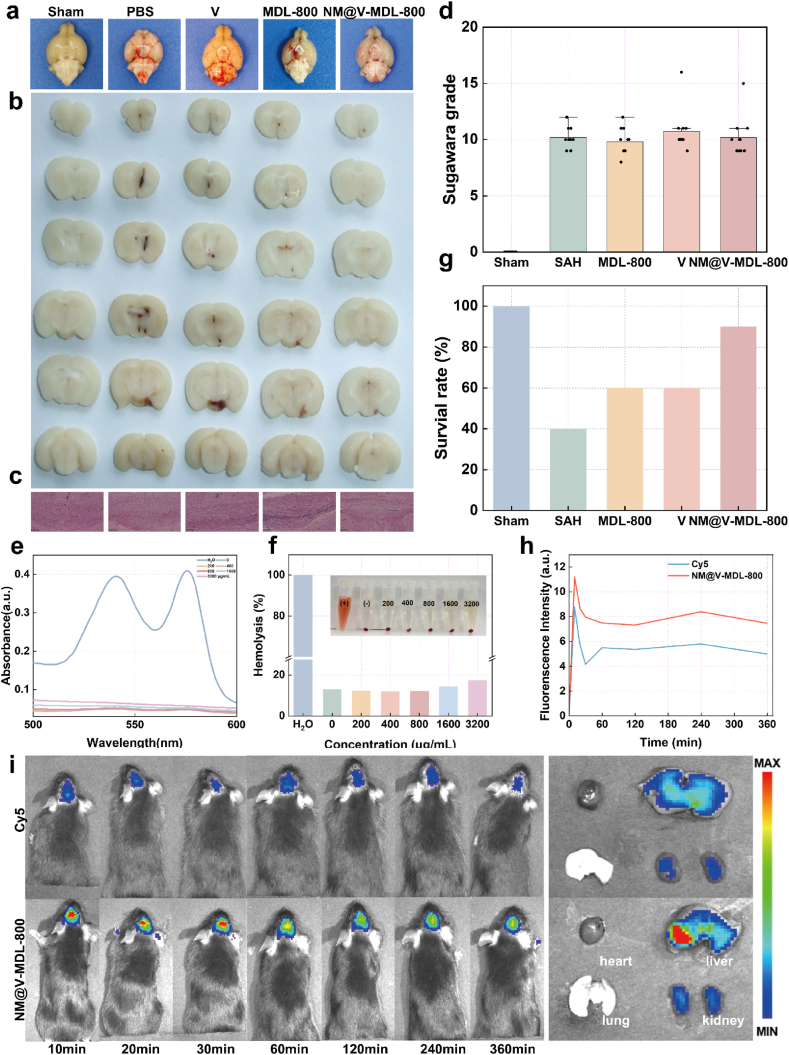
**Biosafety and biodistribution following NM@V-MDL-800.** (a, b) Gross anatomical images and sections of brain tissues from mice in different groups. (c) H&E staining images of brain tissues from mice in different groups. (d) SAH grades of mice in different groups were assessed using the Sugawara method. (e, f) Hemolysis assay and absorbance curve. (g) Survival rates of mice in different groups. (h, i) In vivo fluorescence imaging of the brain, heart, lung, liver, and kidney in mice 10 h after intravenous injection of Cy5-labeled NM@V-MDL-800 and free Cy5, and curves of fluorescence intensity changes in in vivo fluorescence imaging.

To verify the in vivo effect and biosafety of NM@V-MDL-800, we constructed a C57BL/6 mouse SAH model by internal carotid artery puncture [44]. And then established the NM@V-MDL-800 treatment group, free V/SAE treatment group (V), free MDL-800 treatment group, PBS treatment group (SAH), and sham operation group, respectively. Except for the sham operation group, which did not puncture the internal carotid artery, the remaining surgical procedures were the same as those of the other groups. Tail vein injection was performed 1 h after SAH modeling. To ensure the bleeding stability of the SAH model, some mice were sacrificed 72 h after administration. Macroscopic dissection and biopsy observations showed that SAH occurred in all groups except the sham operation group (Fig. 6a and b). The results of brain H&E staining showed that, except for the sham operation group, all mice had red blood cell infiltration in the subarachnoid space. Quantitative results showed that, except for the sham operation group, there was no statistical difference in the proportion of red blood cell infiltration area in the other mice. (Fig. 6c, Fig. S13). In addition, we cited the Sugawara scoring system to assess the bleeding condition of SAH mice [45]. To ensure rigor and consistency, we excluded mice with a bleeding score lower than 8. There was no statistically significant difference in the bleeding degree score among the four groups of patients (Fig. 6d).

The hemolysis test results showed that even at higher concentrations (1600 μg/mL), the hemolysis rate of NM@V-MDL-800 on mice red blood cells was always less than 5 % (Fig. 6e–f). To evaluate the systemic effect of the NM@V-MDL-800 intervention, we analyzed the hematological and biochemical indicators of mice injected with NM@V-MDL-800 on days 1, 2, 7, and 14. The results showed that there was no significant change in blood indicators after the injection of the NM@V-MDL-800 tail vein (Figs. S14–15). The H&E staining results showed no significant damage to major organs such as the heart, liver, and lungs (Fig. S16), further verifying the biological safety of the self-assembled material.

We counted the survival of mice in each group within 72 h. The results showed that the mortality rate of mice within 72 h after SAH was 60 %, but NM@V-MDL-800 significantly reduced the mortality rate (Fig. 6g). To evaluate the biodistribution of NM@V-MDL-800, we labeled NM@V-MDL-800 nanoparticles with Cy5 and injected them into SAH model mice through the tail vein, respectively, with free Cy5 as a control. The live small animal fluorescence imaging technology is adopted to track the fluorescence signals and the distribution of tissues in the body. The results are the fluorescence imaging results at different time points after injection (Fig. 6h–i). Notably, the cerebral fluorescence signal in the NM@V-MDL-800 group was significantly stronger than that in the free Cy5 group, indicating that NM@V-MDL-800 could effectively penetrate the blood-brain barrier and target the inflammatory area after hemorrhage. To further investigate the tissue distribution of NM@V-MDL-800 in mice, we extracted the brain, heart, liver, lungs, kidneys, and other tissues of the mice for fluorescence imaging 10 h after injection. The results showed that compared with the free Cy5 group, stronger fluorescence signals were observed in the liver and kidneys. It is indicated that NM@V-MDL-800 can be effectively metabolized and excreted, which helps to reduce the toxicity of nanozymes in the body. The excellent biological safety and significant targeting ability to NM@V-MDL-800 inflammatory lesion sites provide promising strategies and methods for the treatment of EBI in SAH.

### 2.6 In vivo neuroprotective effects of NM@V-MDL-800

**Fig. 7:**
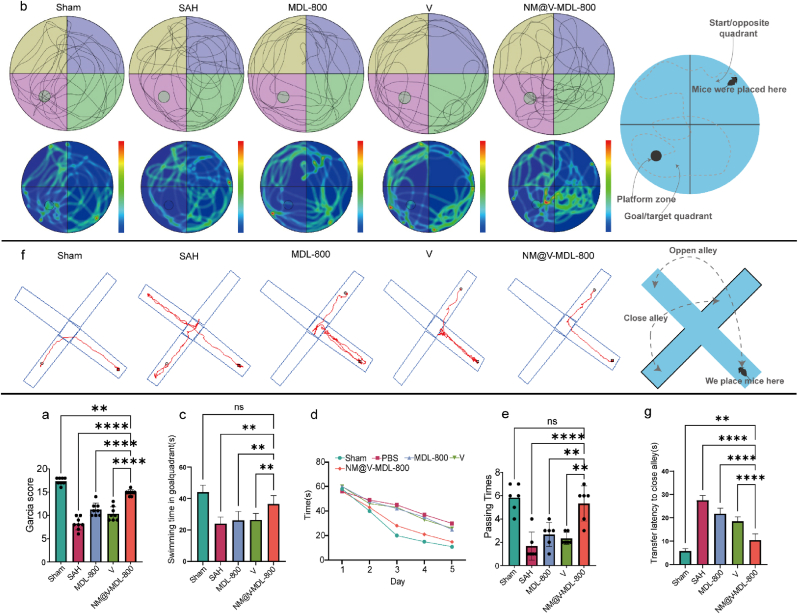
**In vivo neuroprotective effects of NM@V-MDL-800.** (a) Modified Garcia scores of mice in each group at 72 h after SAH induction; (b) Trajectory plots (upper) and heatmaps (lower) of spatial exploration tests showing movement patterns of different treatment groups (n = 7) and schematic; (c) Swimming time in goal quadrant in the Morris water maze test.; (d) Curves of escape latency changes during the 6-day fixed platform training period. (e) Times of crossing the original platform location during the final day^'^s probe trial. (f) Trajectory plots and schematic of elevated plus maze. (g) Time of transfer latency from open alley to close alley.

We evaluated the effects of NM@V-MDL-800 on neural function and memory and cognitive function in SAH model mice using the modified Garcia Neurological Function Score, Morris Water Maze Test (MWM) and elevated plus maze (EPM) [[46], [47], [48], [49]]. The results showed that a significant improvement in the Garcia score was observed at 72 h in the NM@V-MDL-800 treatment group compared to the SAH and V groups and the MDL-800 treatment group (Fig. 7a). In the Morris water maze experiment, 7 mice with hemiplegia were excluded due to the long time spent in the pool and around the pool. The test results of 20 records from 5 platform hidden cycles show that the four groups of animals demonstrated the ability to find platforms on each test day. Compared with the NM@V-MDL-800 group, the deficits in spatial learning ability in the V group, the MDL-800 group, and the SAH group began to emerge on the second day and gradually became obvious in the following four days. In the spatial exploration experiment on the sixth day, the number of platform crossings in the NM@V-MDL-800 group increased significantly, while at the same time no statistically significant difference in swimming distance and swimming speed among the groups (Fig. 7b–e, and S17). Apart from MWM, we have also employed a specific reference memory protocol using the EPM setup, where the time of transfer latency from open alley to close alley was found to be decreased after NM@V-MDL-800 treatment(Fig. 7f and g).The results of neurobehavioral experiments showed that NM@V-MDL-800 had a significant positive effect on the memory and cognitive functions of SAH model mice, which might be closely related to the improvement of early brain injury in SAH mice by NM@V-MDL-800.

**Fig. 8:**
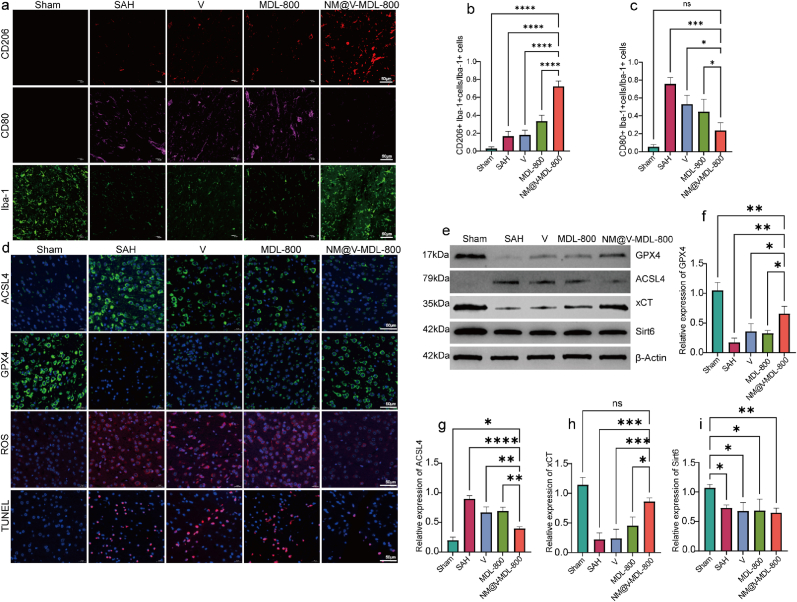
**The effect and mechanism of NM@V-MDL-800 in vivo.** (a–c) Immunofluorescence co-localization staining and corresponding quantification of cortical regions for Iba-1 (green), CD80 (white), CD206 (red), and DAPI (blue). (d) Immunofluorescence staining of cortical regions for ACSL4 (green), GPX4 (green), ROS (red), and TUNEL (red). (e) Western blot analysis of cortical SAH lesion regions. (f–i) Relative expression levels of GPX4, xCT, ACSL4, and Sirt6. ∗∗∗P < 0.001, ∗∗P < 0.01, ∗P < 0.05. (For interpretation of the references to color in this figure legend, the reader is referred to the Web version of this article.)

To further investigate the effect of NM@V-MDL-800 on microglial polarization in SAH mouse models, immunofluorescence staining was used to detect the expression levels of microglial markers Iba-1, M2 phenotypic marker CD206, and M1 phenotypic marker CD80 in brain slices in the SAH cortex injury area (Fig. 8a–ce). The results showed that the number of CD80-positive cells in the hemorrhagic injury area increased significantly after SAH occurred, indicating that SAH could induce M0 microglia to activate the M1 state. After treatment with NM@V-MDL-800, the proportion of CD206-positive cells was significantly increased, and the number of CD80-positive cells decreased accordingly. This result shows that NM@V-MDL-800 can promote the polarization of microglia from M1 to M2 in the SAH model, effectively blocking the vicious cycle of ferroptosis and neuroinflammation caused by M1 polarization.

Given that phenotypic transformation of microglia is strongly associated with ferroptosis processes, we then tested the MDA level, and the results shown that, similar to the in vitro experiment, the MDA level in brain tissue increased significantly after SAH and decreased significantly after adding NM@MDL-800(Fig. S18). And further performed TUNEL staining experiments and immunofluorescence analysis for ferroptosis-related markers on brain injury slices of SAH mouse models (Fig. 8d, Figures S19-22). The experimental results showed that the SAH group had the highest level of cell death, while the number of cell deaths in the NM@V-MDL-800 treatment group was significantly reduced. In immunofluorescence staining experiments, NM@V-MDL-800 significantly increased the expression level of GPX4, which was significantly better than that of the V group and the MDL-800 treatment group. In contrast, the expression levels of ACSL4 and ROS were significantly increased in the SAH group, but decreased to a level close to that of the sham group after treatment with NM@V-MDL-800, and the efficacy was significantly better than that of the V group and MDL-800 group. The expression levels of xCT, ACSL4, GPX4, and Sirt6 were further evaluated by Western blot (WB) analysis (Fig. 8e–i) [[50], [51], [52], [53]]. RT-qPCR experimental results show that after adding the ferroptosis inducer RSL3, the effect of NM@V-MDL-800 on microglial cells polarizing to M2 was reversed (Fig. S23). The results of the analysis were consistent with in vitro experiments, confirming that NM@V-MDL-800 could effectively inhibit ferroptosis in SAH model mice, thereby disrupting the harmful cycle associated with the transformation of microglia to the M1 phenotype.

## 3 Conclusion

In summary, this study successfully developed a neutrophil membrane-encapsulated V/SAE-polyphenol self-assembly biomimetic nanozyme, which improves early brain injury after subarachnoid hemorrhage by inhibiting the key pathophysiological process of ferroptosis. The V-MDL-800 consists of MDL-800 and V/SAE,with the help of neutrophil membrane encapsulation, it is given the ability to cross the BBB and target sites of the subarachnoid hemorrhagic neuroinflammatory response. In vitro studies have shown that NM@V-MDL-800 can effectively scavenge ROS, activate the Sirt6/xCT/GPX4 pathway, reduce microglial oxidative stress and lipid metabolism disorders, prevent microglial ferroptosis by improving lipid peroxidation, and induce its polarization from M1-like to M2-like isoforms, ultimately alleviating neuroinflammation. In vivo experiments further confirmed that NM@V-MDL-800 successfully crosses the BBB and targets aggregation in the lesion area, significantly improves survival rate, and enhances memory and cognitive function, which are inseparable from the inhibition of the ferroptosis pathway and the regulation of microglial polarization, and has fewer adverse effects. This study proposes a new therapeutic strategy, namely the application of nano-encapsulated biomimetic single-atom nanozymes, which provides a new perspective for the nanomedical treatment of SAH targeting microglial ferroptosis, and has broad application prospects.

## CRediT authorship contribution statement

**Boliang Liu:** Conceptualization. **Chao Xiang:** Conceptualization. **Xiaodan Zhang:** Formal analysis. **Wei Guo:** Investigation. **Haitao Wu:** Investigation. **Fandi Hou:** Formal analysis. **Yueyang Ba:** Investigation. **Xiulei Zhang:** Investigation. **Zhongcan Chen:** Resources. **Guang Feng:** Software. **Yuan Dang:** Supervision. **Yang Zhu:** Writing – review & editing, Writing – original draft, Supervision. **Jianjun Gu:** Supervision.

## Availability of data and materials

Data is provided within the manuscript or supplementary information files.

## Ethical approval/informed consent

Animal experiments were performed according to the protocol (IACUC FJMU2022-0608) approved by the Ethical Committee of Fujian Medical University.

## Declaration of competing interest

The authors declared that they have no known competing financial interests or personal relationships that could have appeared to influence the work reported in this paper.

## Data Availability

Data will be made available on request.
