# Supplementary material for: Inflammation-targeted single-atom nanozymes drive microglial depolarization and inhibit ferroptosis via Sirt-6-xCT-GPX4 axis to attenuate early brain injury following subarachnoid hemorrhage
Source: Mater Today Bio. 2026 Jan 20;37:102829. doi: 10.1016/j.mtbio.2026.102829 (PMC12859461; doi:10.1016/j.mtbio.2026.102829)
Supplement: Multimedia component 1 [file mmc1.docx]

**Inflammation-targeted Single-Atom Nanozymes Drive Microglial Depolarization and Inhibit Ferroptosis via Sirt-6-xCT-GPX4 Axis to Attenuate Early Brain Injury Following Subarachnoid Hemorrhage**

**Materials and methods**

1. **Materials and Reagents**

Hydrogen peroxide (H_2_O_2_) was obtained from Sinopharm Chemical Reagents (Shanghai, China). Cy5 was obtained from YuanYe Bio-Technology (Shanghai, China).C11-BODIPY581/591 were provided by Sigma-Aldrich (St. Louis, USA). Hoechst 33342, 2′,7′-dichlorofluorescin diacetate (DCFH-DA), cell counting kit-8 (CCK-8), AM/PI, and 1,1',3,3'-tetraethyl-5,5',6,6'-tetrachloroimidacarbocyanine iodide (JC-1) were acquired from Beyotime (Shanghai, China). Electron microscopy fixatives were obtained from Bergolin Biotechnology (Dalian, China). Antibodies against Glutathione Peroxidase 4 (GPX4), Anti-FACL4 (ACSL4), H3K9ac，beta Actin, and secondary antibodies goat anti-rabbit were sourced from Abcam (Cambridge, UK). CD40, CD80, Arginase (Arg)-1 and CD206 were obtained from Thermo Fisher Scientific (Waltham, USA); Antibodies against xCT，Sirt6 were procured from Cell Signaling Technology (Danvers, MA, USA). Antibodies against H3K56ac and Histone H3 were procured from Huabio（Hangzhou，China). Deionized (DI) water was penetrated using a Milli-Q water purification system.

1. **Instrumentation**

The X-ray diffraction (XRD) patterns of the powder were recorded on the Miniflex-600 diffractometer of Rikko Corporation in Japan. The transmission electron microscope (TEM) images were captured by Hitachi-7700. High-angle annular dark field scanning transmission electron microscopy (HAADF-STEM) images were recorded using a JEM-ARM200F (JEOL) TEM/STEM with a spherical aberration corrector. Energy-dispersive X-ray spectroscopy (EDS) mapping was performed by JEM-2100F. X-ray photoelectron spectroscopy (XPS) spectra were collected on the scanning X-ray microprobe (PHI 5000 Verasa, ULAC-PHI). The scanning electron microscopy (SEM) images were captured by Nova NanoSEM 230. Fluorescence imaging was achieved using a Zeiss 800 confocal laser scanning microscope (CLSM). The luminescence data were collected by the SYNERGY H1 microplate reader from Biotek.

1. **Synthesis of NM@V-MDL-800**

V@ZIF-8 precursor was synthesized through the host-guest strategy. The preparation method of Solution A is to add 3.54 g of 2-methylimidazole and 100 mg of V(acac)₃ to a 150 mL flask,which contains 60mL of methanol. Solution B is formed by dissolving 1.6 g of Zn(NO₃)₂·6H₂O in 60mL of methanol. After mixing, solutions A and B were stirred for 12 hours. The resulting V@ZIF-8 powder was collected by centrifugation, washed with methanol, and dried overnight in a vacuum oven at 65°C. The V@ZIF-8 powder was heated at 950°C for 3 hours in an argon atmosphere in a tube furnace and subjects to one-step pyrolysis to obtain V-N₄/SAN.

When preparing NM@V-MDL-800, mix 10 mg of V-N₄/SAE with 1 mL of 1 mg/mL MDL-800 (dissolved in DMSO) and sonicate for 1 hour. Centrifuged the mixture, washed it three times with PBS. Subsequently, 1.5 mL of 1 mg/mL neutrophil membranes (NM) was added, and the mixture was processed with a liposome extruder to obtain NM@V-MDL-800.

1. **Evaluation of SOD-like Activity of NM@V-MDL-800**

The experimental procedure involves preparing NM@V-MDL-800 in concentrations of（eg.25, 50, 100, 150, 200）μg/mL in the assay buffer. In a 96-well microplate, 20 μL of each sample or standard is mixed with 200 μL of WST working solution. After adding 20 μL of enzyme working solution to initiate the reaction, the plate is incubated at 37 °C for 30 min in a humidified environment. Absorbance at 450 nm was measured using the Synergy H1 microplate reader.

1. **Assessment of CAT-like Activity of NM@V-MDL-800**

NM@V-MDL-800 all use concentrated series, such as 0，50，100，200μg/mL Phosphate-buffered saline (PBS, pH 7.4). Calibrate the YSI 5100 dissolved oxygen meter equipped with a Clark-type oxygen electrode according to the manufacturer’s instructions. Then, add 2 mL of each nanoparticle suspension to a sealed reaction chamber with a temperature controller set to 25 ± 0.1 °C. Rapidly inject 20 μL of 30% H₂O₂ (final concentration: 100 mM) into the chamber to initiate the reaction. Oxygen evolution was continuously monitored for 5 min, with data points recorded every 5 s.

1. **Evaluation of POD-like Activity of NM@V-MDL-800 Nanoparticles**

Hydroxyl radicals were generated via the Fenton reaction using FeSO₄·7H₂O and H₂O₂. In a 96-well microplate, the following reagents were added sequentially: 50 μL of FeSO₄·7H₂O solution (1.8 mM), 50 μL of NM@V-MDL-800 nanoparticle suspension at various concentrations, 50 μL of salicylic acid solution (3 mM), and 50 μL of H₂O₂ solution (0.03%). The reaction mixture was then incubated at 37 °C in the dark for 30 min. The absorbance was measured at 510 nm using a Synergy H1 microplate reader to evaluate the results.

1. **DPPH Radical Scavenging Assay**

The antioxidant activity of the sample was evaluated using the DPPH (1,1-diphenyl-2-picrylhydrazyl) radical scavenging assay. DPPH is a stable nitrogen-centered free radical whose ethanol solution exhibits a purple color and strong absorption at 517 nm. The scavenging effect of antioxidants on DPPH radicals results in a decrease in solution absorbance, which is proportional to the radical scavenging ability of the sample. A stock solution of DPPH (0.1 mM) was prepared in ethanol. The sample solutions were diluted to various concentrations using ethanol. For the assay, 100 μL of the sample solution was mixed with 100 μL of DPPH solution in a 96-well plate. The mixture was shaken gently and left to stand at room temperature for 30 minutes in the dark. The absorbance was measured at 517 nm using a microplate reader. The DPPH radical scavenging activity was calculated using the formula: Scavenging activity (%)=(*A*control​−*A*sample​​)/ *A*control​×100.where *A*control​ is the absorbance of the DPPH solution without the sample, and *A*sample​ is the absorbance of the DPPH solution with the sample. The experiment was performed in triplicate, and the results were expressed as the mean ± standard deviation.

1. **Intracellular Uptake and Lysosomal Co-localization of Cy5-Labeled NM@V-MDL-800**

Cy5-labeled NM@V-MDL-800 was prepared by conjugating with Cy5 NHS ester for purification. BV-2 cells were incubated with the nanozymes at different time points, and lysosomes were stained with LysoTracker Green. After washing, the cells were imaged using a Zeiss LSM 800 confocal microscope, and images were analyzed with ZEN Blue software and ImageJ. The co-localization of Nanozyme-lysosome was assessed through the JACoP plugin, and the Manders overlap coefficient and Pearson correlation coefficient were calculated.

1. **In Vitro Model of SAH and Experimental Group Design**

BV-2 cells were induced to simulate SAH by supplementing hemoglobin (10 mM) to the culture medium. Subsequently, the cells were divided into five groups: 1.Control group (Vehicle): cells cultured under normal conditions; 2.Hemin group (Hemin): cells were incubated in medium supplemented with heme (10 mM) for 12 hours; 3.Free MDL-800 group (MDL-800): cells in the heme group treated with free MDL-800 for 12 hours; 4.Free V/SAE group (V): cells in the hemin-induced group treated with free V/SAE for 12 hours; 5.NM@V-MDL-800 group (NM@V-MDL-800): cells in the hemin-induced group treated with NM@V-MDL-800 for 12 hours. Select the above-mentioned time point as the best opportunity to observe morphological changes, conduct functional assessments, and evaluate the effects of drug interventions.

1. **In Vitro Cytotoxicity Assessment**

Cell cytotoxicity was evaluated using the Cell Counting Kit-8 (CCK-8). Hemin-induced cells were inoculated in 96-well plates. Cells were treated with NM@V-MDL-800, free V/SAE and free MDL-800 (0, 12.5, 25, 50, 100, 200 μg/mL) for 24 h. At the end of the treatment, 10 μL of CCK-8 solution was added to each well, and the cells were incubated at 37°C for 2 hours in a humidified environment containing 5% CO2. The absorbance at 450 nm was measured using a SYNERGY H1 microplate reader. According to the CCK-8 guidelines, the relative viability of the treated cells compared to the untreated cells was calculated to assess cytotoxicity and protective effects. Live/dead staining was used to directly visualize the cytotoxicity of the five groups of cells.

1. **Viability Staining of Live/Dead Cells**

BV-2 cells were seeded at a density of 1×10⁵ cells per well in polylysine-coated confocal dishes. After drug treatment, the cells were gently washed twice with PBS and incubated in HBSS buffer containing 2 μM Calcein-AM and 4 μM PI. The cells were incubated in the dark at 37 °C for 30 minutes to allow the dye to penetrate fully. Imaging was performed using an LSM 980 confocal microscope system: live cells (green fluorescence) were detected under 488 nm excitation, and dead cells (red fluorescence) were detected under 561 nm excitation. The ratio of green fluorescence intensity to red fluorescence intensity was calculated using ImageJ software to evaluate cell mortality.

1. **Apoptotic Cell Evaluation**

The apoptosis rate of BV2 cells was assessed by annexin V-fluorescein isothiocyanate (FITC)/propidium iodide (PI) Apoptosis Kit(V13242;Thermo Scientific,Eugene,American) Cells were digested with trypsin and then suspended (1 × 106 cells) in 500 µL of 1× binding buffer. The 5 µL of Annexin V-FITC staining solution and 10 µL of PI staining solution were introduced, mixed properly, and incubated at 37 °C for 5 min. The percentage of apoptotic cells was calculated using FlowJo for quantification.

1. **In Vitro Reactive Oxygen Species (ROS) Detection**

The intracellular ROS levels in the four groups were quantitatively determined using the 2′,7′-dichlorodihydrofluorescein diacetate (DCFH-DA) fluorescent probe. The cells were inoculated in confocal dishes and co-incubated with 10 µM DCFH-DA and Hoechst for 30 minutes at 37°C. After incubation, the cells were washed with phosphate-buffered saline (PBS) to remove excess probe. Fluorescence intensity reflecting ROS production was measured using a confocal laser scanning microscope (CLSM) at an excitation wavelength of 488 nm and an emission wavelength of 525 nm.

1. **Detection of Mitochondrial Membrane Potential**

The mitochondrial membrane potential of BV-2 cells was quantitatively assessed using the JC-1 probe. JC-1 is a voltage-sensitive, lipophilic cationic fluorescent dye that specifically accumulates in the mitochondrial matrix. Under normal mitochondrial membrane potential, JC-1 forms J-aggregates emitting red fluorescence; when the membrane potential decreases, it remains in monomeric form and emits green fluorescence. The ratio of red and green fluorescence intensities accurately reflects the functional state of mitochondria.

The JC-1 working solution was prepared in serum-free medium with a final concentration of 2 μM. After removing the original culture medium from each group of cells, the JC-1 working solution pre-warmed (37 °C) was added to the culture dish. Then incubate the Petridis in a dark incubator at 37 °C for 15 minutes, with gentle shaking 2–3 times during incubation to ensure even distribution of the dye. After incubation, the cells were carefully washed three times with JC-1 staining buffer to remove unbound dye molecules. Fluorescence imaging was performed using a Zeiss LSM 980 confocal microscope. Green fluorescence (monomer) was excited at 488 nm and detected at 530 ± 15 nm emission; Red fluorescence (aggregates) was excited at 543 nm and detected at 590 ± 15 nm emission.

1. **Transmission electron microscopy (TEM) Reveal mitochondrial architecture**

Cells were seeded in 6-well plates. After treatment with corresponding drugs, with PBS washing cells, 3 % with butyl glycol (fixed liquid) fixed under 4℃ for 2 h. After removal of the fixative, the cells were immersed in PBS twice for 10 min each. Subsequently, they were fixed with 1 % osmium tetrachloride for 1 h. After removing the fixative solution, the cells underwent two additional immersions in PBS for a duration of 10 min each time. Dehydration was performed using acetone/isoamyl acetate (1:1) for 10 min, followed by further dehydration using isoamyl acetate for 30 min. Electron micrographs were captured using an 80kV Hitachi HT7800 transmission electron microscope (HITACHI, Japan).

1. **In Vitro** **Lipid Peroxidation (LPO) Assay**

The LPO levels in five cell groups were evaluated using C11-BODIPY581/591, which was a fluorescent probe that shifts from red fluorescence to green fluorescence upon oxidation. Cells were inoculated in confocal culture dishes and incubated with C11-BODIPY581/591 and Hoechst at an optimal concentration (10 μM) for detection. After treatment, cells were washed with PBS to remove all unbound dyes. The shift of fluorescence was monitored using CLSM to capture images of the oxidized and reduced forms of the probe were captured at appropriate excitation and emission wavelengths. The ratio of green (∼510 nm) to red (∼590 nm) fluorescence was calculated, providing a quantitative measurement of intracellular LPO.

1. **Malondialdehyde (MDA) level assessment**

MDA assay kit (BC6410‐50T/48S) was from Solarbio, Beijing, China. All measurements were performed according to manufacturer's instructions.

1. **In Vitro Protein Expression Analysis**

The protein levels of H3K9ac, H3K56ac, Sirt6, GPX4, ACSL4 and xCT were detected by Western blotting. Cells from five experimental groups (control group, Hemin, free MDL-800, free V/SAE and NM@V-MDL-800) were harvested and mixed with protease inhibitors PMSF (Beyotime, China). Total proteins were extracted using the ExKine™ Pro Animal Cell/Tissue Total Protein Extraction Kit (AbbKine, USA). The PVDF membranes were blocked with 5% skimmed milk and then incubated overnight at 4°C with specific primary antibodies against H3K9ac, H3K56ac, Sirt6, GPX4, ACSL4 and xCT. After washing, the PVDF membranes were incubated with HRP-conjugated secondary antibodies. The protein bands were visualized using an enhanced chemiluminescence (ECL) detection system. The Band strength was quantified by densitometry and normalized to β-actin and H3 as load controls.

1. **Animal Grouping and Treatment**

Animal experiments were performed according to the protocol (IACUC FJMU2022-0608) approved by the Ethical Committee of Fujian Medical University.

Male C57BL/6J mice aged 10-12 weeks (Beijing Vital River) were used. All mice were raised in a specific pathogen-free (SPF) environment with the temperature of 23-25°C and a humidity of 55-60%, and maintained on a standard diet under a 12:12-hour light/dark cycle. The subarachnoid hemorrhage (SAH) model was established using the arterial puncture method. Non-absorbable 4-0 polypropylene suture (W8557, Ethicon, USA) was applied to puncture the right internal carotid artery (ICA). The sham-operated mice underwent the same procedure, except that the filament was advanced 3 mm into the right ICA without puncture. During the operation, a heating pad kept the body temperature at 37°C ± 0.5°C.

To evaluate the effect of NM@V-MDL-800 on mice after SAH, the animals were randomly divided into 5 groups: Sham group (Sham): Sham; SAH group (SAH): Intravenous injection of PBS (0.01 ml/g) via the tail vein 1 hour after SAH; Free V/SAE group (V): Intravenous injection of free V/SAE (0.01 ml/g) via the tail vein 1 hour after SAH; Free MDL-800 treatment group (MDL-800): Intravenous injection of free MDL-800 at the same concentration via the tail vein 1 hour after SAH; NM@V-MDL-800 group; (NM@V-MDL-800): Intravenous injection of NM@V-MDL-800 at the same concentration via the tail vein 1 hour after SAH.

1. **SAH Severity Score and Neurological Function Score**

The SAH classification is evaluated based on the Sugawara method. The animal is euthanized 72 hours after drug intervention, and the brain tissue is dissected and removed. The basal carpool is divided into six parts, and each part is assigned a score from 0 to 3 based on the degree of blood in the subarachnoid space: 0 indicates no SAH; 1 indicates mild SAH; 2 represents moderate SAH, and 3 represents severe SAH. Add up the scores of all parts to get the total score ranging from 0 to 18. Mice showing mild SAH (total score ≤7 points) were excluded from subsequent studies.

The neurological function of the five groups of mice 72 hours after SAH was evaluated by the modified Garcia scoring method. Neurological deficits were evaluated on a total score of 0 to 18 based on six tests: spontaneous activity (in a cage for 5 minutes), spontaneous limb movement, forelimb extension (when holding the tail), barbed wire climbing, response to touch on the side of the trunk, and response to touch on the beard. The higher the score, the better the neurological function.

1. **Hemolysis Assay**

The hemolytic potential of NM@V-MDL-800 was evaluated using mouse blood. Blood samples were collected from the orbital sinus of mice; the fresh blood samples were incubated with various concentrations of NM@V-MDL-800 (0, 200, 400, 800, 1600, 3200 μg/ml), and treated with H2O and PBS as the negative control groups. After incubation, the samples were centrifuged, and the absorbance of the supernatant was measured at 540 nm to quantify the degree of hemolysis. The hemolysis rate (%) was calculated using the following formula: Hemolysis rate (%) = [(OD experimental - OD negative control) / (OD positive control - OD negative control)] × 100%, with an optical density of 540 nm.

1. **In Vivo Imaging and Drug Distribution in Brain Tissue**

NM@V-MDL-800 was labeled with Cy5 and free Cy5 was used as a control. Subsequently, the labeled compounds were injected into the SAH model mice through the tail vein. Fluorescence images were captured at 10 min, 20 min, 30 min, 1 h, 2 h, 4 h, and 6 h after injection using the PerkinElmer IVIS Lumina III system.

1. **Histopathological Staining, Hematological, and Biochemical Analyses**

To evaluate the systemic effects of NM@V-MDL-800 intervention, weekly hematological and biochemical analyses were conducted on mice injected with NM@V-MDL-800 for 14 days. Blood samples were collected at predetermined time points through posterior orbital venous plexus or tail vein puncture to detect complete blood count (CBC) parameters (including white blood cells, neutrophils, lymphocytes, monocytes, red blood cells, and hemoglobin) and biochemical indicators (liver function: ALP, ALT, AST; kidney function: creatinine).

After 14 days of drug intervention, the mice were sacrificed, and heart, liver, spleen, lung, kidney, and brain tissues (SAH lesion area) were collected. The tissue samples were fixed with 4% paraformaldehyde and embedded in paraffin to prepare 5-micron slices. Hematoxylin-eosin (H&E) staining was used to evaluate the morphological integrity and pathological changes (such as neuronal degeneration, inflammation, or necrosis). The brain tissues of mice that participated in the experiment after the EBI time window were excluded from subsequent analyses.

1. **Behavioral Experiments**

Before the grouping operation and SAH modeling (day 0), all animals underwent a comprehensive behavioral screening to ensure consistent baseline levels. Only animals with normal behavioral parameters were included in the study and randomly assigned to the experimental groups.

The water maze test recording and analysis instrument (Zhongshi Technology, China) was used to assess spatial learning and memory using the modified Morris water maze (MWM) method. The training began 72 hours after the operation. From days 1 to 5, set a fixed time slot for each day, and train four times in each time slot. At the beginning of the training, the platform was placed in the NW quadrant, and the mice were placed in the pool facing the wall from any of the four starting points on the pool wall. The video recording system recorded the time it took for the mice to find the platform (escape latency) and their swimming paths. During the four training sessions, the mice were respectively placed in the water from four different starting points (different quadrants). If the platform is found or not found within 60 seconds (the latency was recorded as 60 seconds), the experimenter will guide the mice to find the platform, rest on it for 10 seconds, and then conduct the next experiment. The average of the incubation periods of the mice during four training sessions each day was taken as the mice's academic performance for that day. On the 6th day, remove the original platform and place the mice at any one entry point into the water. All mice must be at the same entry point. Record the number of times the rats cross the position of the original platform within 2 minutes. Mice that showed prolonged immobility (immobility time > 5 seconds for two consecutive tests) during the formal test were considered unsuitable for the water maze test (possibly due to severe hemiplegia) and were excluded from the study. At least six mice in each experimental group were ultimately guaranteed to complete the test.

The elevated plus maze was used to assess reference memory- like behavior. This behavior is based on the approach-avoidance conflict in rodents in response to elevation. Animals have a natural tendency to escape from an open alley of EPM to an enclosed alley. Mice were placed individually at the end of either of the open arms, and the duration the animal took to move from the open to the enclosed arm (TL) was noted on the 1st day. The animals were allowed to explore the apparatus for 60 s. On the next day, after the first exposure, TL was again noted. The training lasted three days and record the time of transfer latency from open alley to close alley of fourth day. The apparatus was properly cleaned between the two trials to avoid the influence of previous animals

**20. Brain tissue processing and immunofluorescence detection**

72 hours after drug intervention, the mice were anesthetized and euthanized: first, they were perfused with pre-cooled physiological saline, then fixed with paraformaldehyde, and subsequently kept overnight in the fixative. Immunofluorescence staining was used to detect the expression of CD206/Iba-1, CD80/Iba-1, SLC7A11, GPX4, ACSL4, ROS and TUNEL. After blocking, the sections were incubated with the primary antibodies specific to each protein, and then the secondary antibody was fluorescently labeled. Fluorescence was detected using a confocal microscope.

**21. 1. Protein expression analysis of brain tissue**

The protein levels of Sirt6, GPX4, ACSL4, and xCT were detected by Western blotting. Cut the tissue, weigh it, with each sample weighing approximately 100mg, then lysis with 1ml of RIPA cell lysis buffer (containing 1mM PMSF). The samples were centrifuged at 12,000rpm for 10 minutes, and the collected supernatant, which contained the total protein of the brain tissue. The subsequent experimental methods were the same as those in the cell experiments.

**22. 1. Statistical Analysis**

All quantitative data are presented as mean ± standard deviation (SD). Statistical analysis was performed using GraphPad Prism 10.1.2 software. For experiments requiring statistical analysis, at least three independent experiments were conducted (n ≥ 3). Paired t-tests were used for two groups of data, and one-way ANOVA was used for multiple groups. Statistical significance was indicated as *P < 0.05, **P < 0.01, ***P < 0.001, ****P < 0.0001. Unless otherwise stated, all comparisons were made relative to the control group.

**
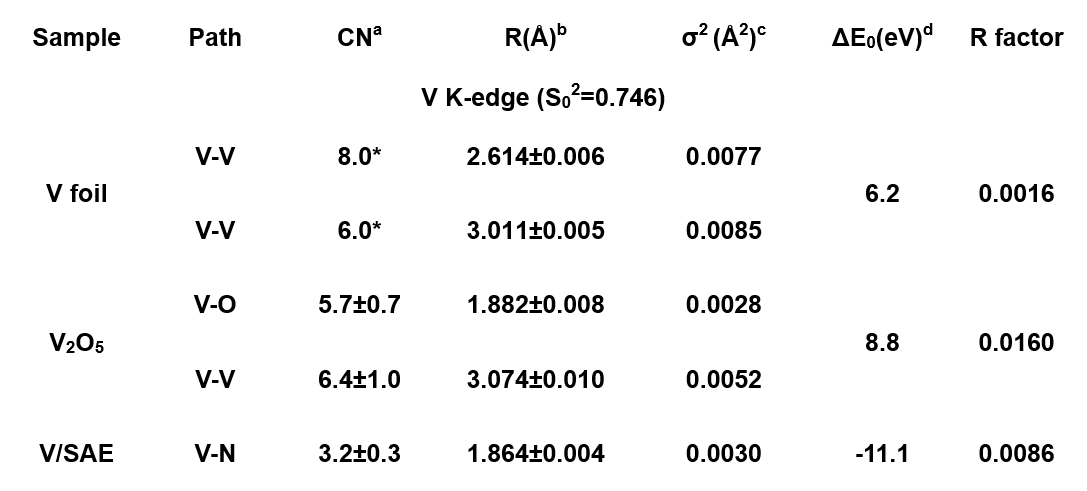
**

**Table S1. EXAFS data fitting results of Samples.** *^a^CN*, coordination number; *^b^R*, the distance between absorber and backscatter atoms; *^c^σ*^2^, the Debye Waller factor value; *^d^ΔE*_0_, inner potential correction to account for the difference in the inner potential between the sample and the reference compound; *R* factor indicates the goodness of the fit. *S*0^2^ was fixed to 0.746, according to the experimental EXAFS fit of V foil by fixing *CN* as the known crystallographic value. * This value was fixed during EXAFS fitting, based on the known structure of V. Fitting conditions: *k* range：2.0 - 94.0; *R* range: 1.0-2.5; fitting space: R space; *k*-weight = 3. A reasonable range of EXAFS fitting parameters: 0.800 < *Ѕ*_0_^2^ < 1.000; *CN >* 0; *σ*^2^ > 0 Å^2^; |Δ*E*_0_| < 15 eV; *R* factor < 0.02.

**
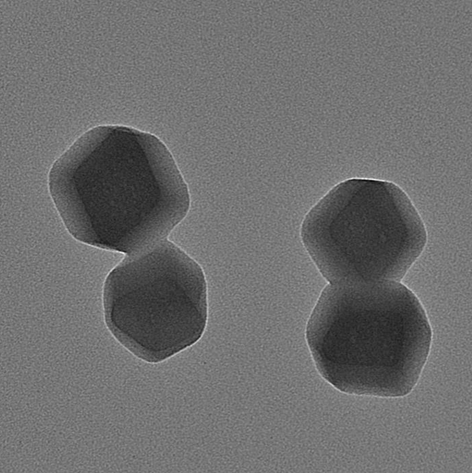
**

**Figure S1. TEM image of V@ZIF-8**

**
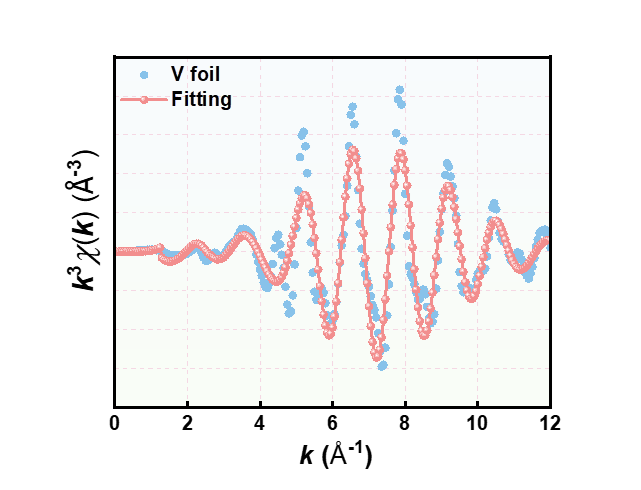
**

**Figure S2. EXAFS fitting curves of V foil at the k space.**

**
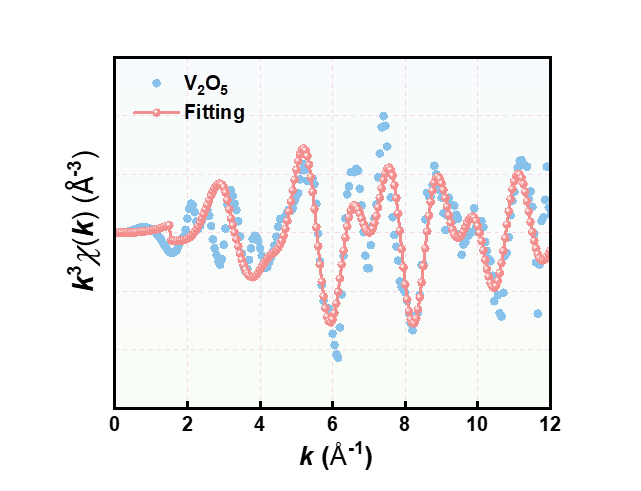
**

**Figure S3. EXAFS fitting curves of V_2_O_5_ at the k space.**

**Figure S4. Wavelet transformation of V K-edge EXAFS of V_2_O_5_.**

**
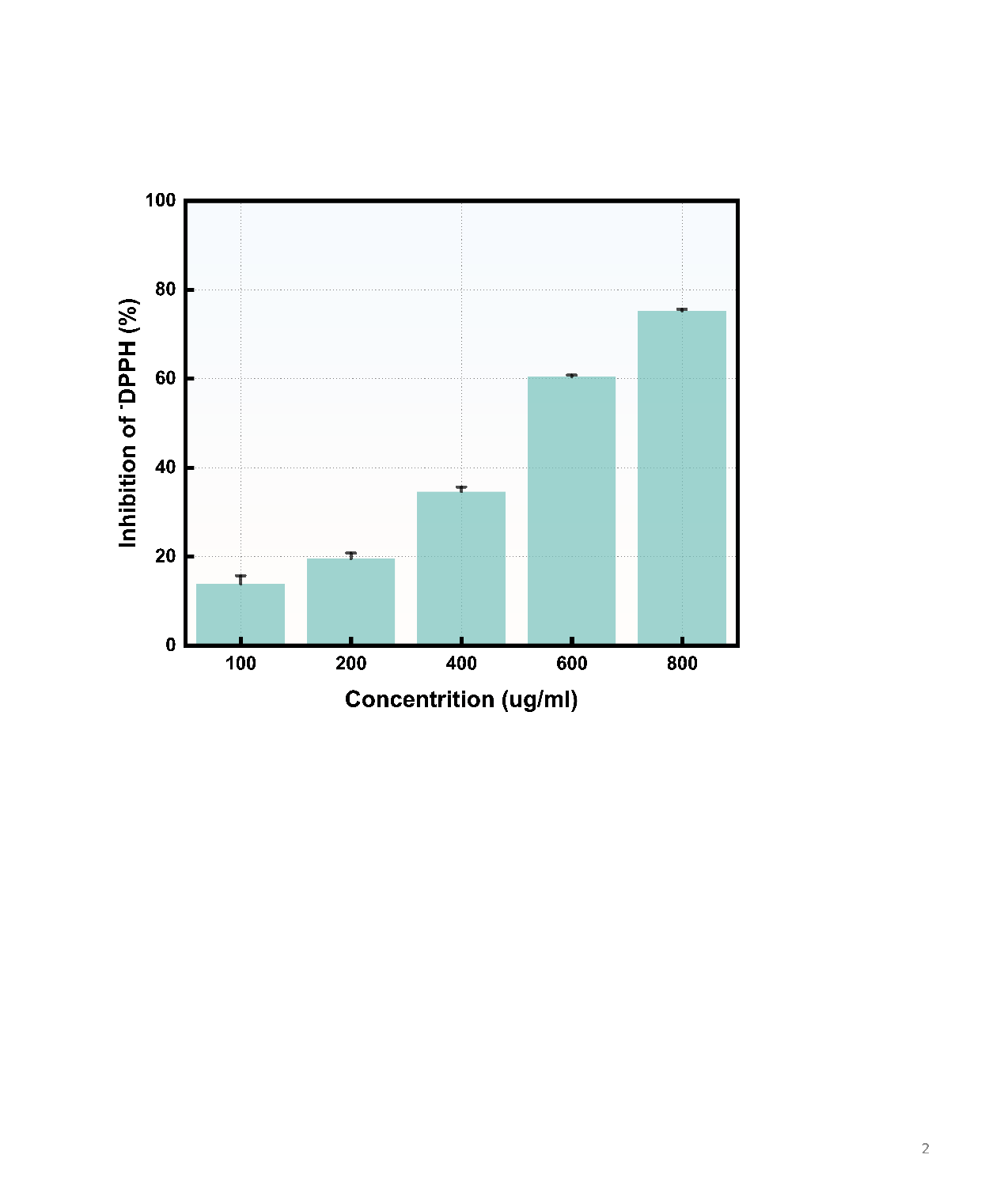
Figure S5. Evaluate the ROS scavenging ability of NM@V-MDL-800 at different concentrations using the DPPH assay kit.**

**
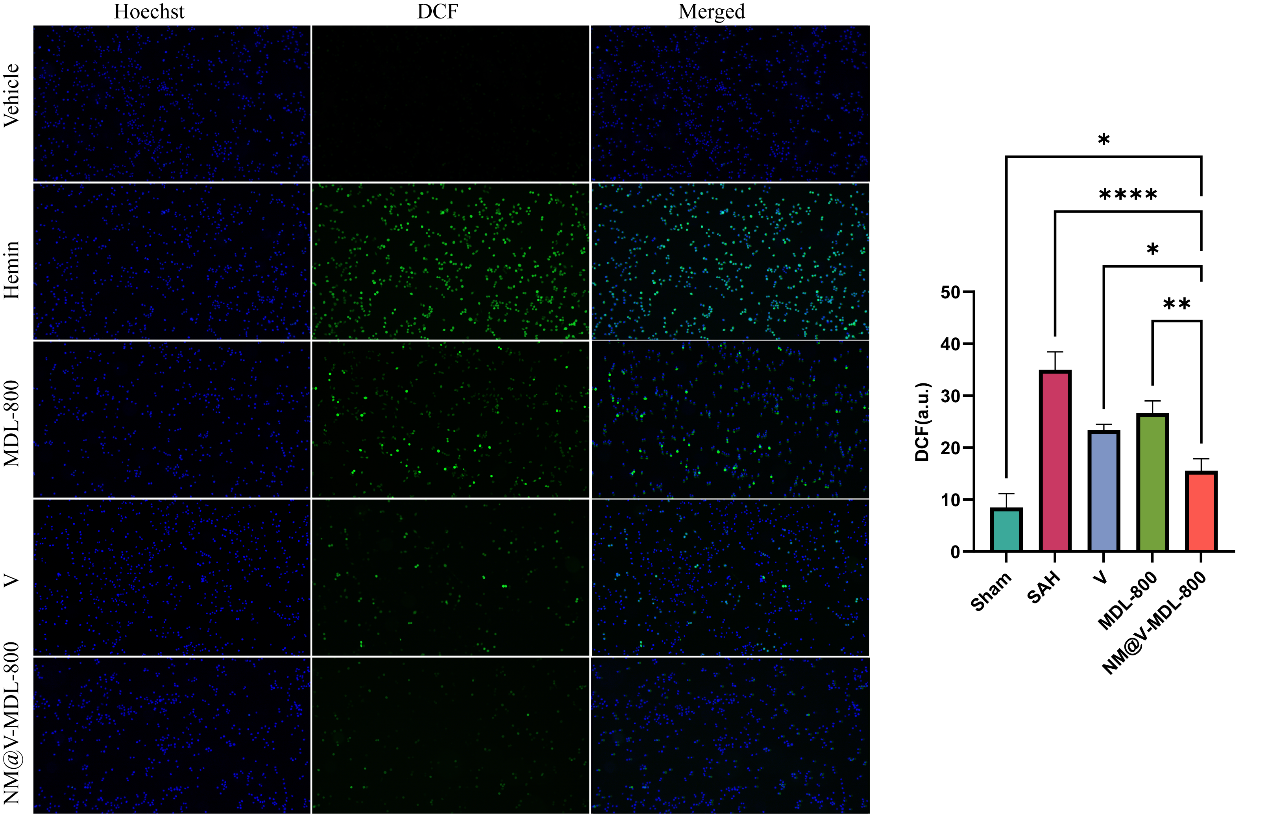
**

**Figure S6. Intracellular production of reactive oxygen species (ROS) and the average fluorescence intensity corresponding to quantification by CLSM following various treatments (DCFH-DA as the probe). ***P＜0.001. **P＜0.01. *P＜0.05.**


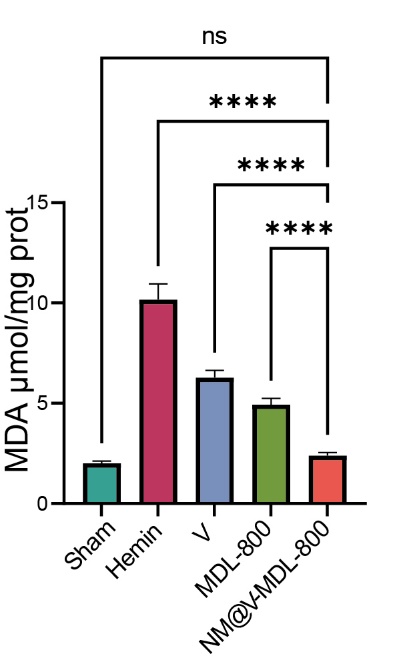


**Figure S7. The MDA levels were measured by commercial assay kits in cellular.**

**Figure S
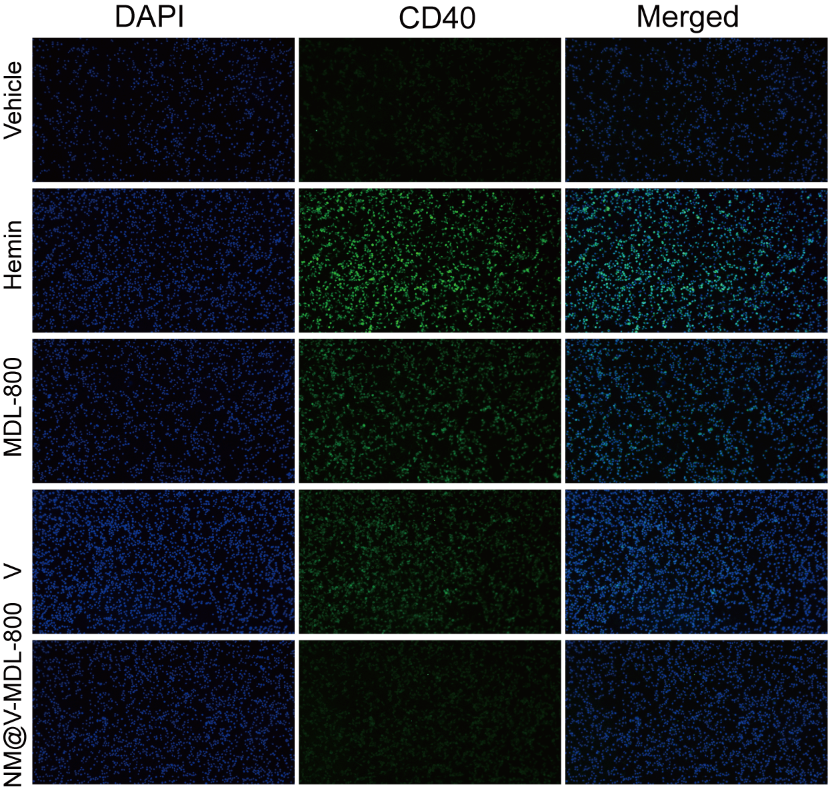
8. CLSM images of fluorescence markers for CD40.**


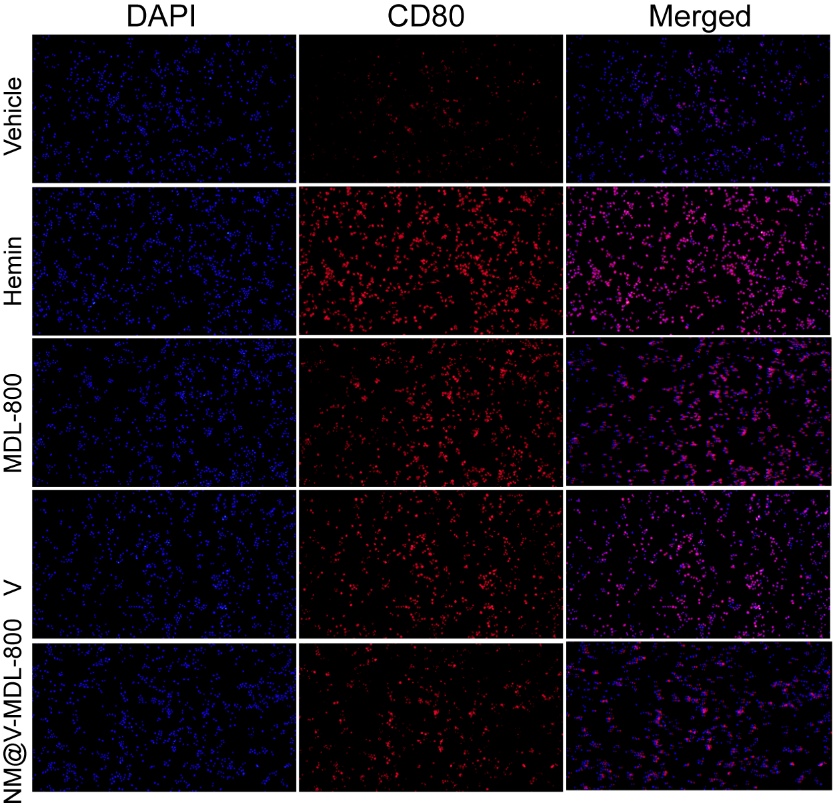


**Figure S9. CLSM images of fluorescence markers for CD80.**


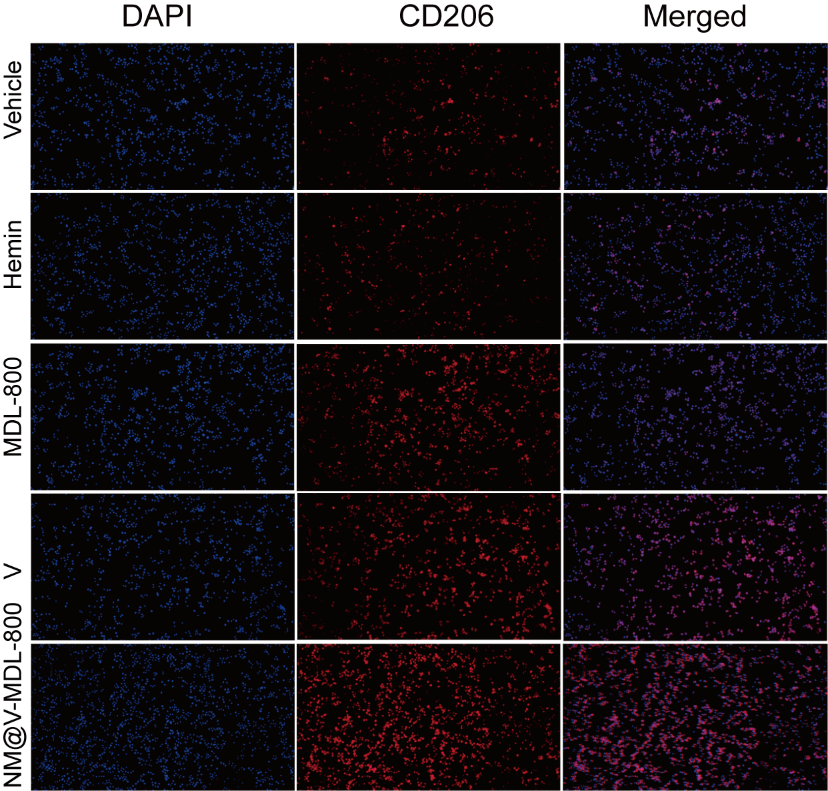


**Figure S10. CLSM images of fluorescence markers for CD206.**


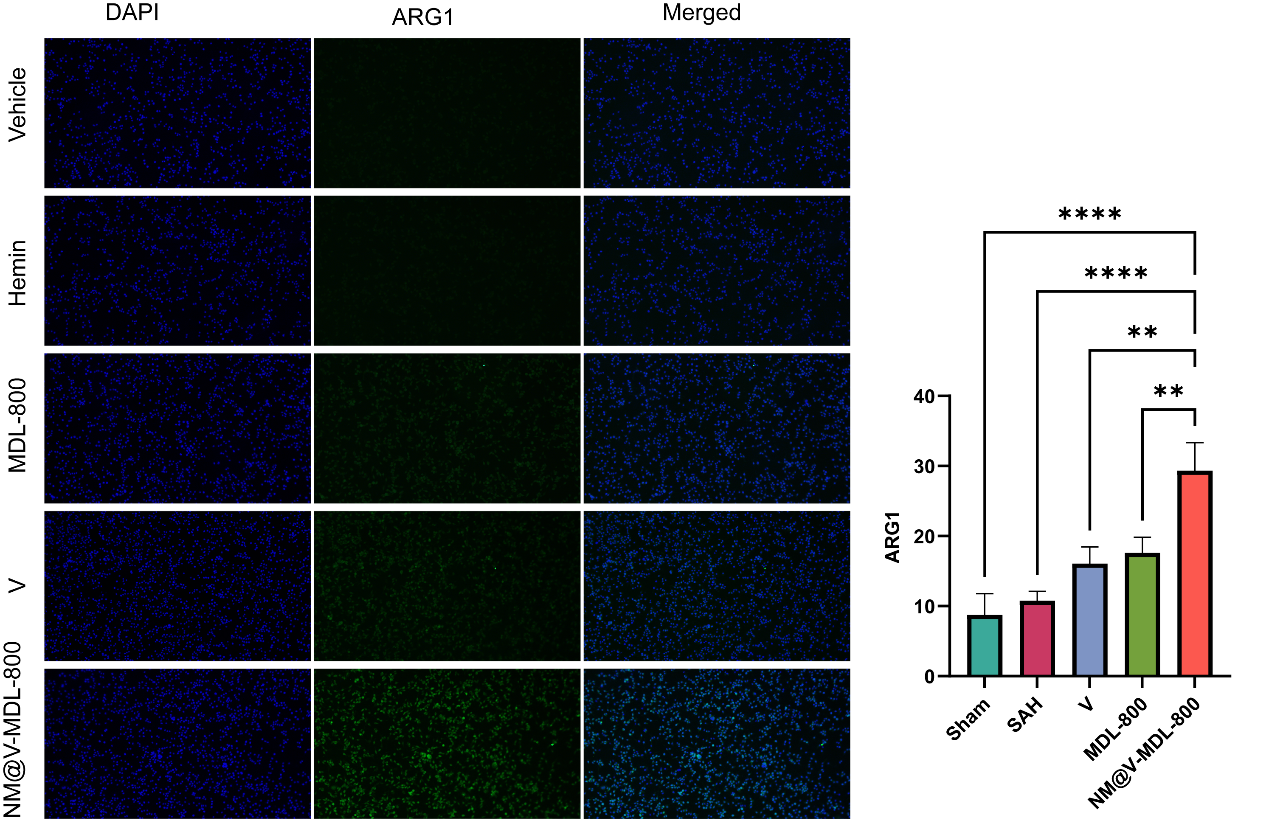


**Figure S11. CLSM images of fluorescence markers for ARG1.**


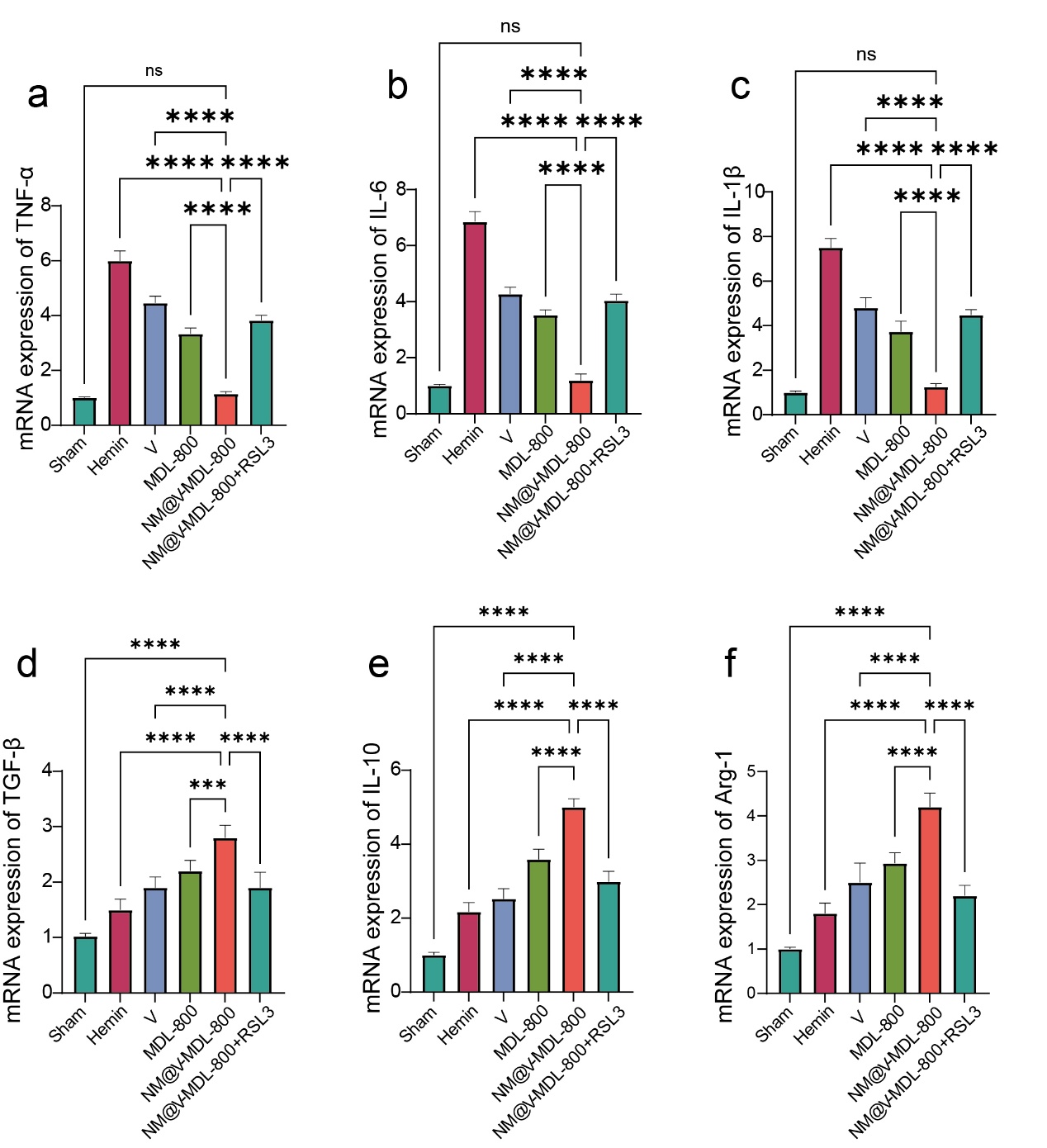


**Figure S12. The qPCR assay indicating the mRNA expression levels of TNF-a, IL-6, IL-1β, TGF-β, IL-10 and Arg-1 in vitro.**


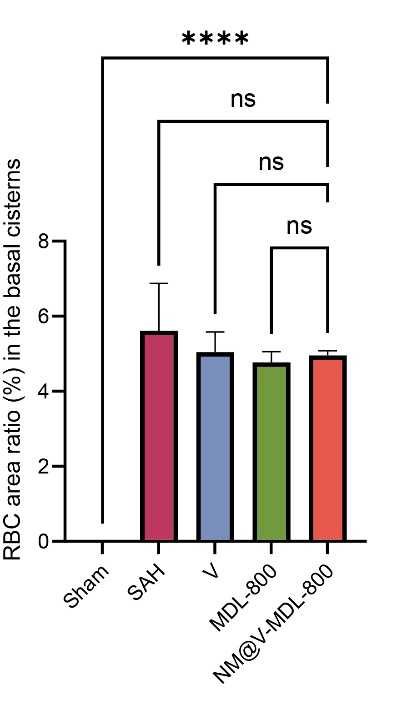


**Figure S13. Quantitative analysis of the area ratio of red blood cells in brain tissue HE staining.**

**
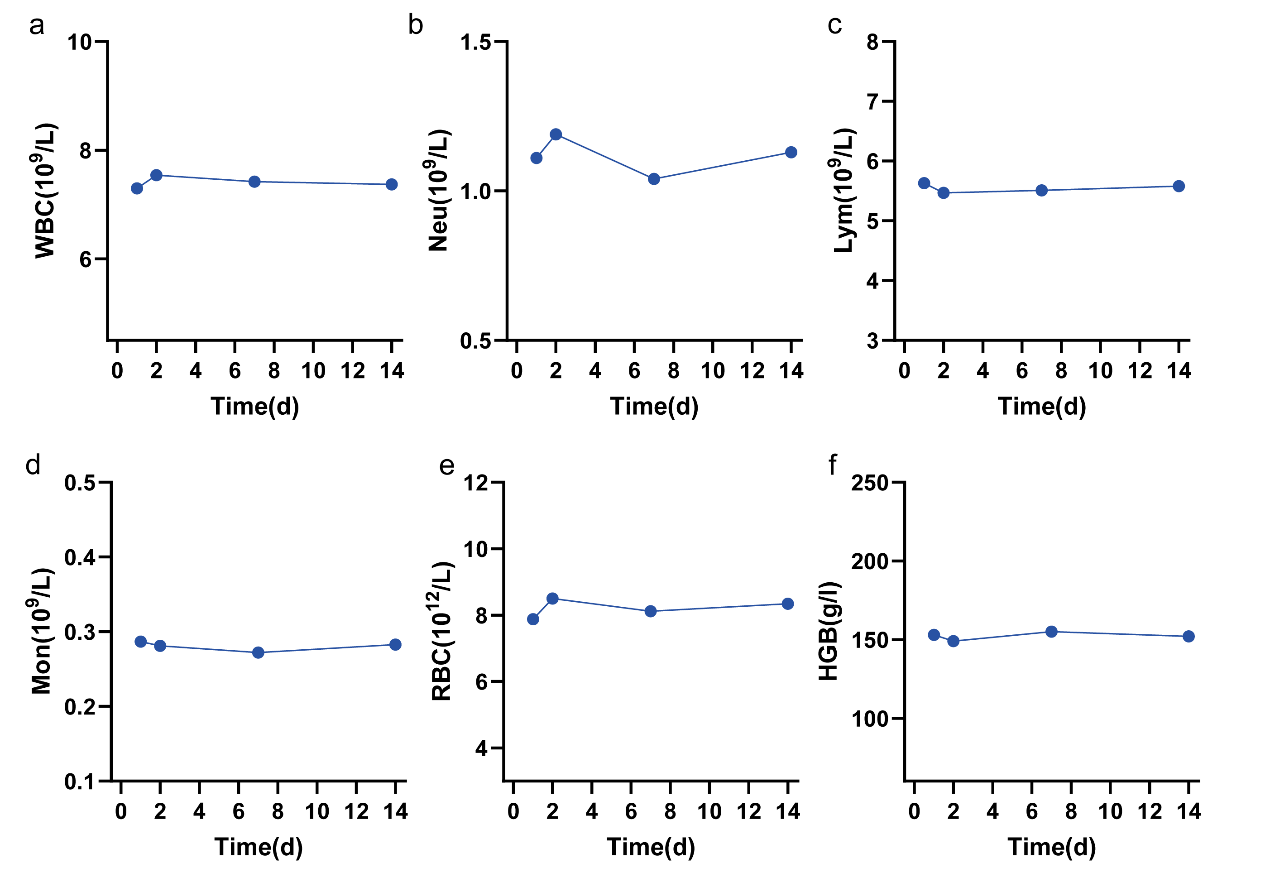
**

**Figure S14. Blood routine examination in mice at day 1, 2, 7, and 14 post-injection of NM@V-MDL-800. (a)** **WBC, white blood cell; (b) Neu, neutrophil; (c) Lym, lymphocyte; (d) Mon, monocyte; (e) RBC, red blood cell; (f) HGB, hemoglobin.**

**
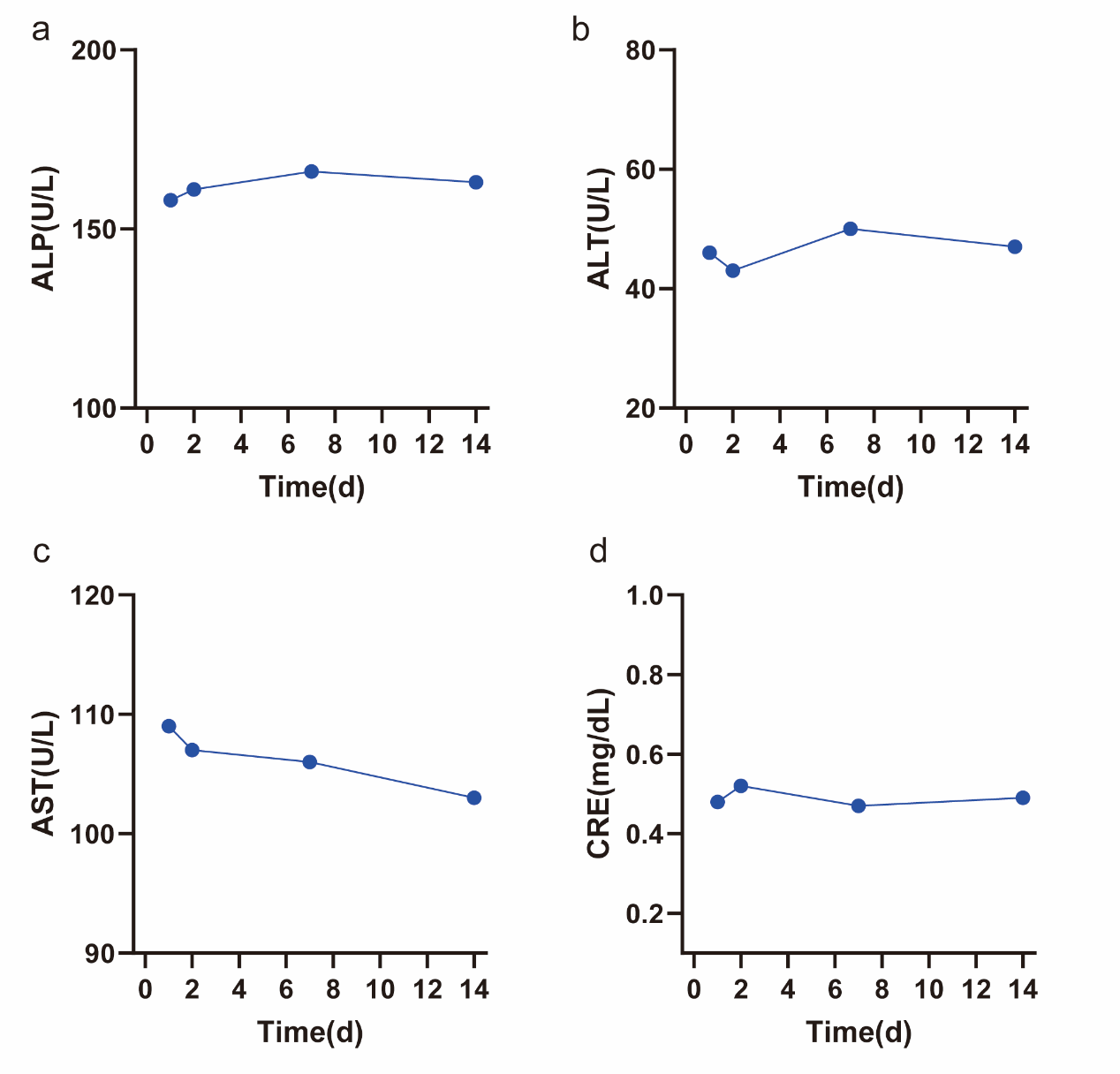
**

**Figure S15. Blood biochemistry analysis in mice at day 1, 2, 7, and 14 post-injection of NM@V-MDL-800. (a) Alkaline phosphatase (ALP); (b) Alanine transaminase (ALT); (c) Aspartate aminotransferase (AST); (d) Creatinine (CRE).**


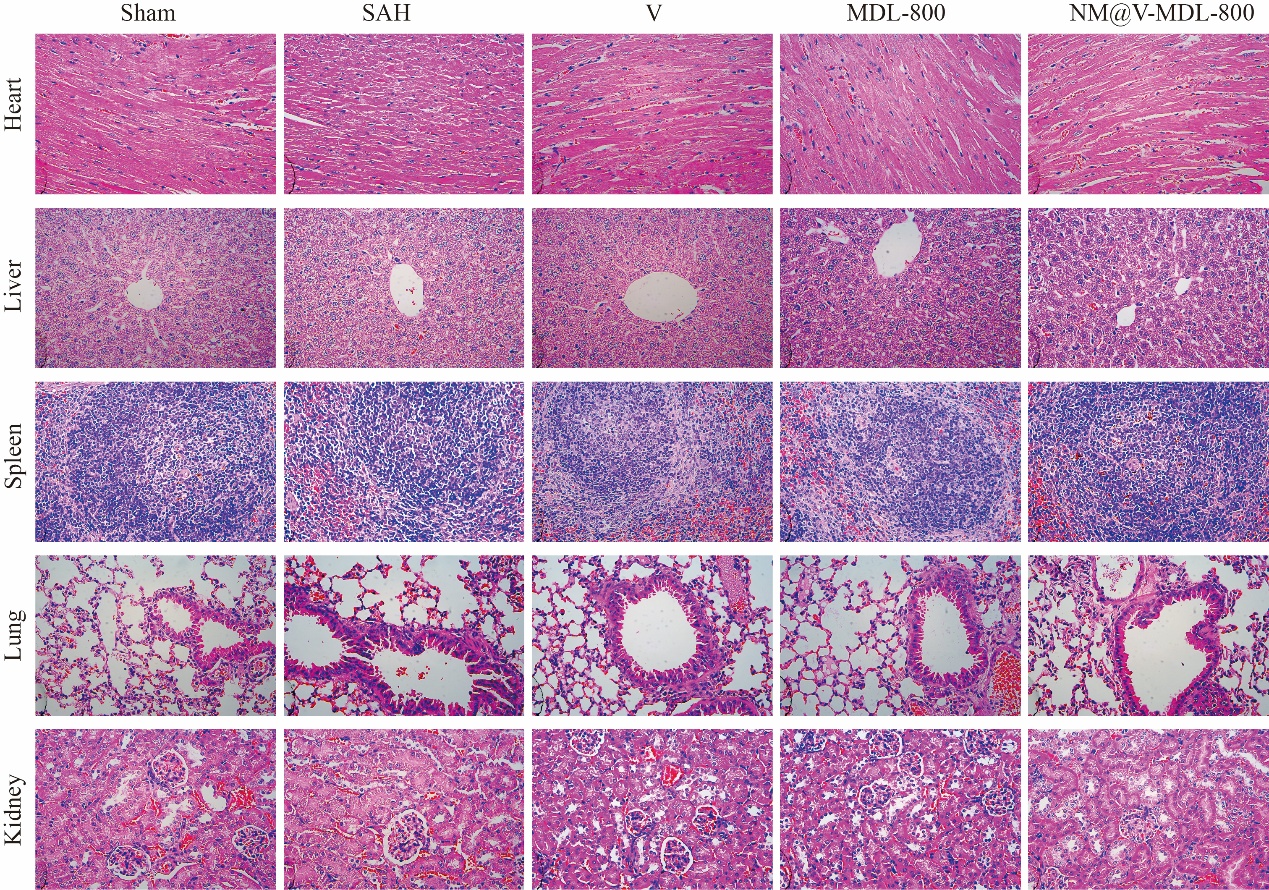


**Figure S16. H&E-stained images of major organs harvested from different groups of mice at 14 days post-treatment.**

**Figure S1**
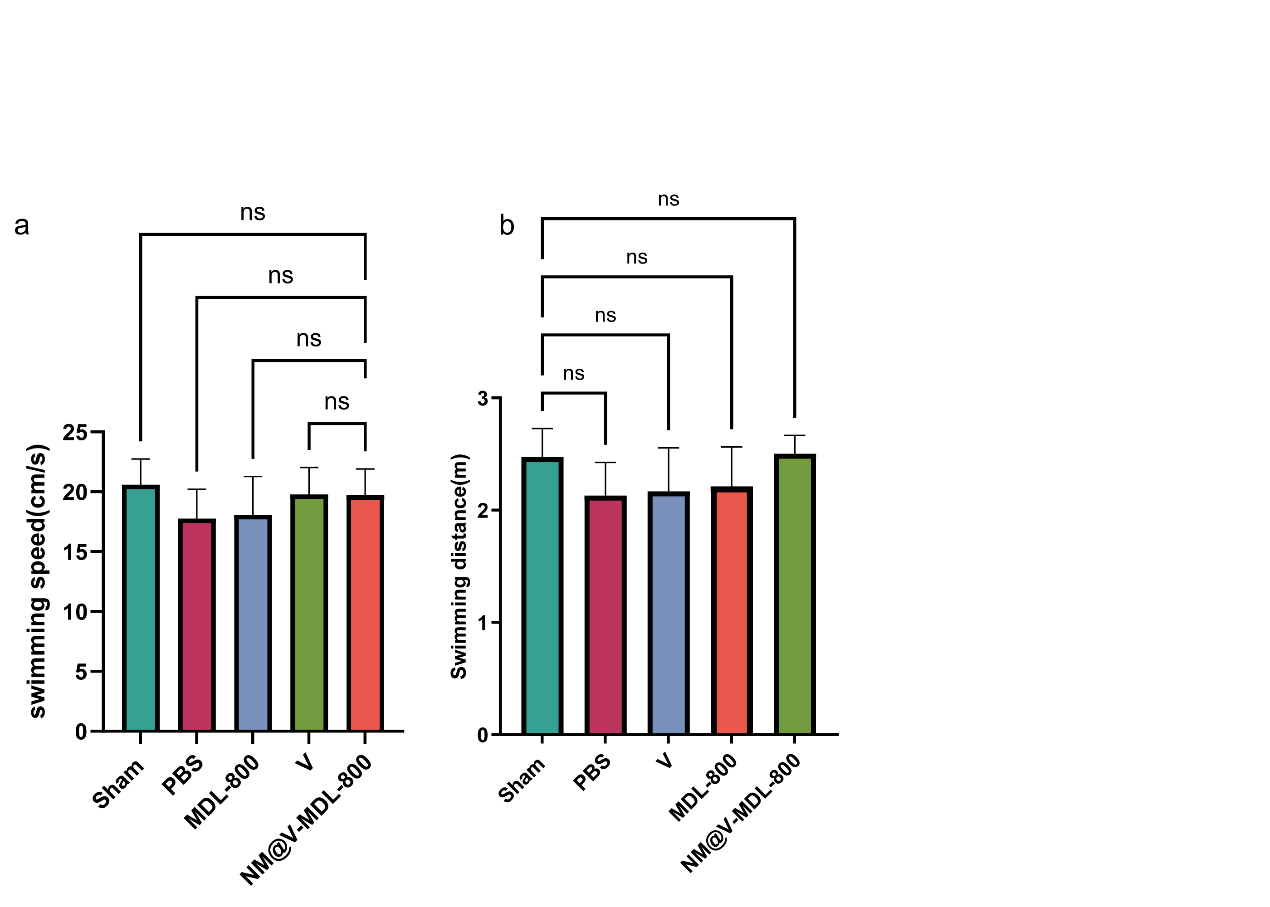
**7. Swimming speed(a) and swimming distances(b) during the final day’s probe trial of morris water maze trials**


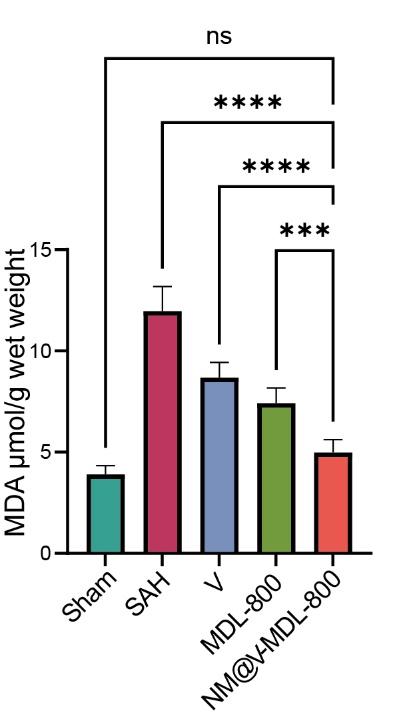


**Figure S18. The MDA levels were measured by commercial assay kits in brain tissue samples.**


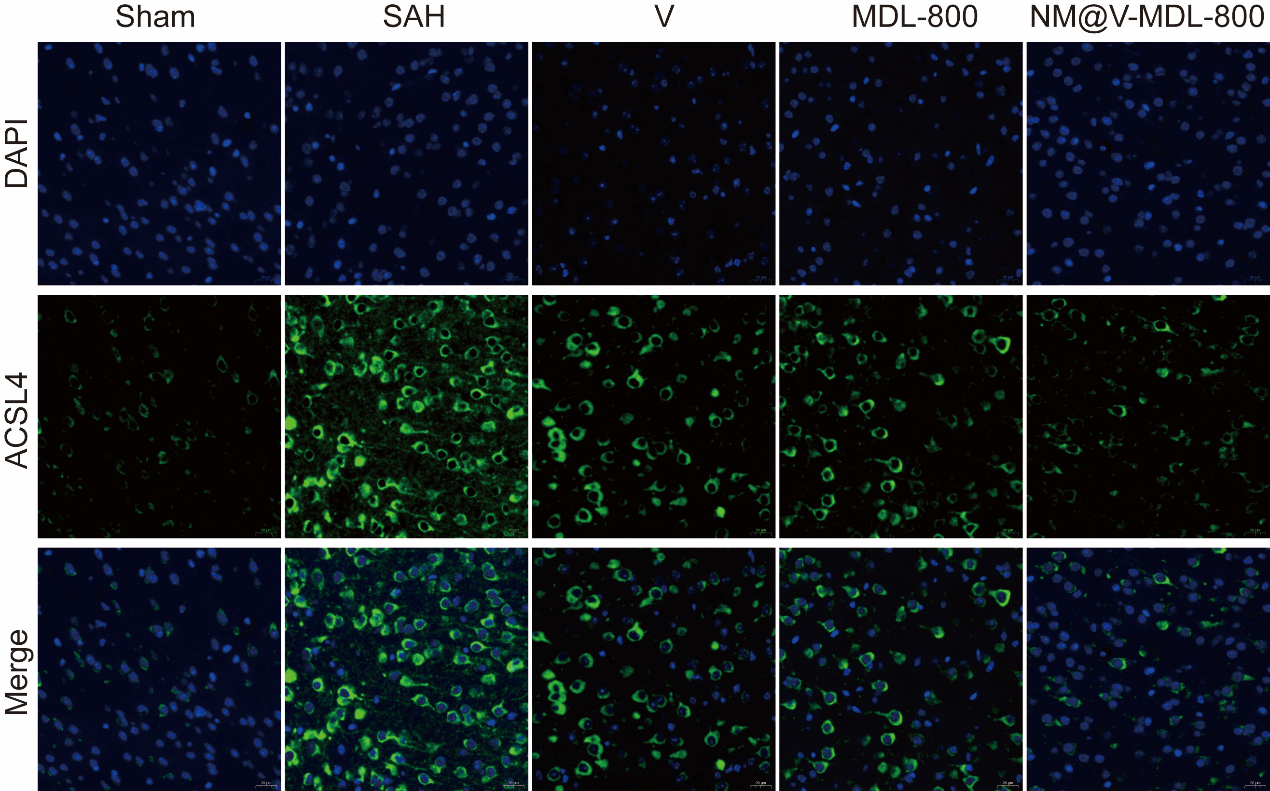


**Figure S19.** **Co-immunofluorescence staining of ACSL4 (green) and DAPI (blue) in the SAH lesion area.**


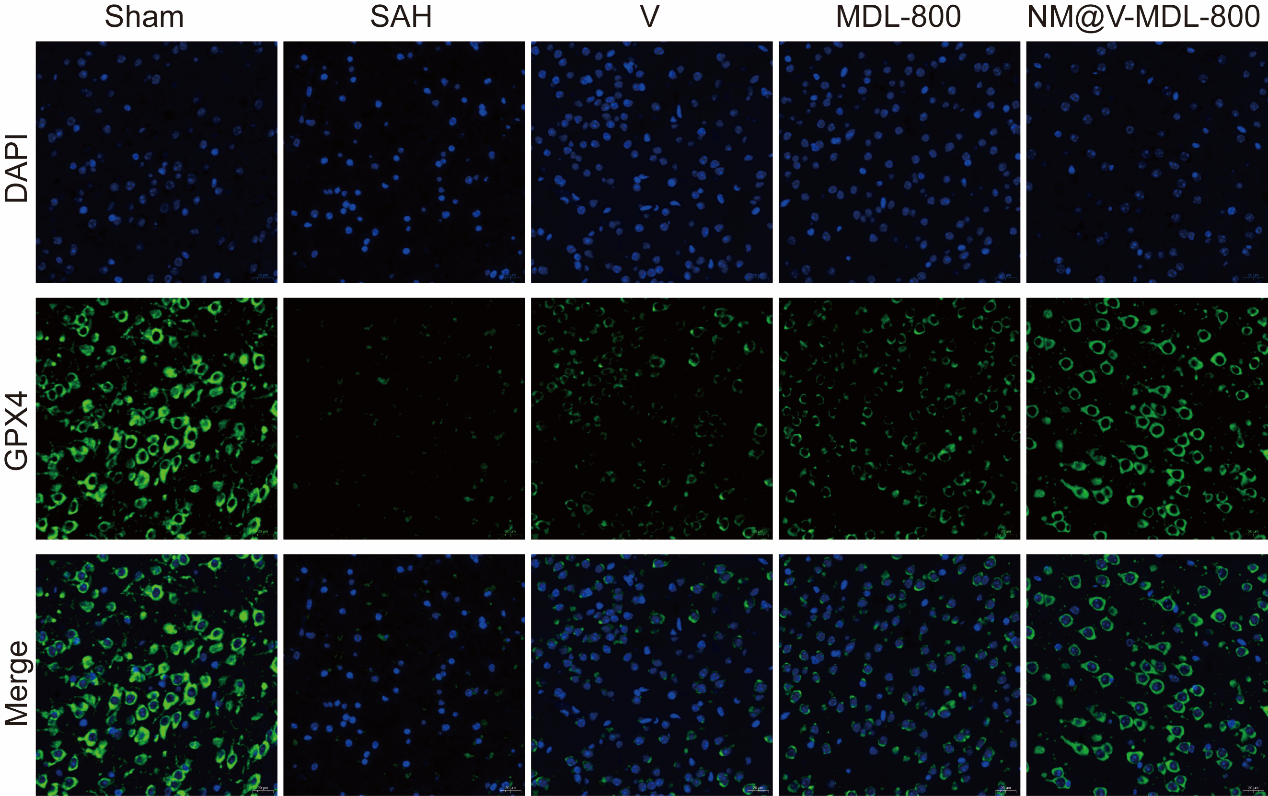


**Figure S20**. **Co-immunofluorescence staining of GPX4 (green) and DAPI (blue) in the SAH lesion area.**


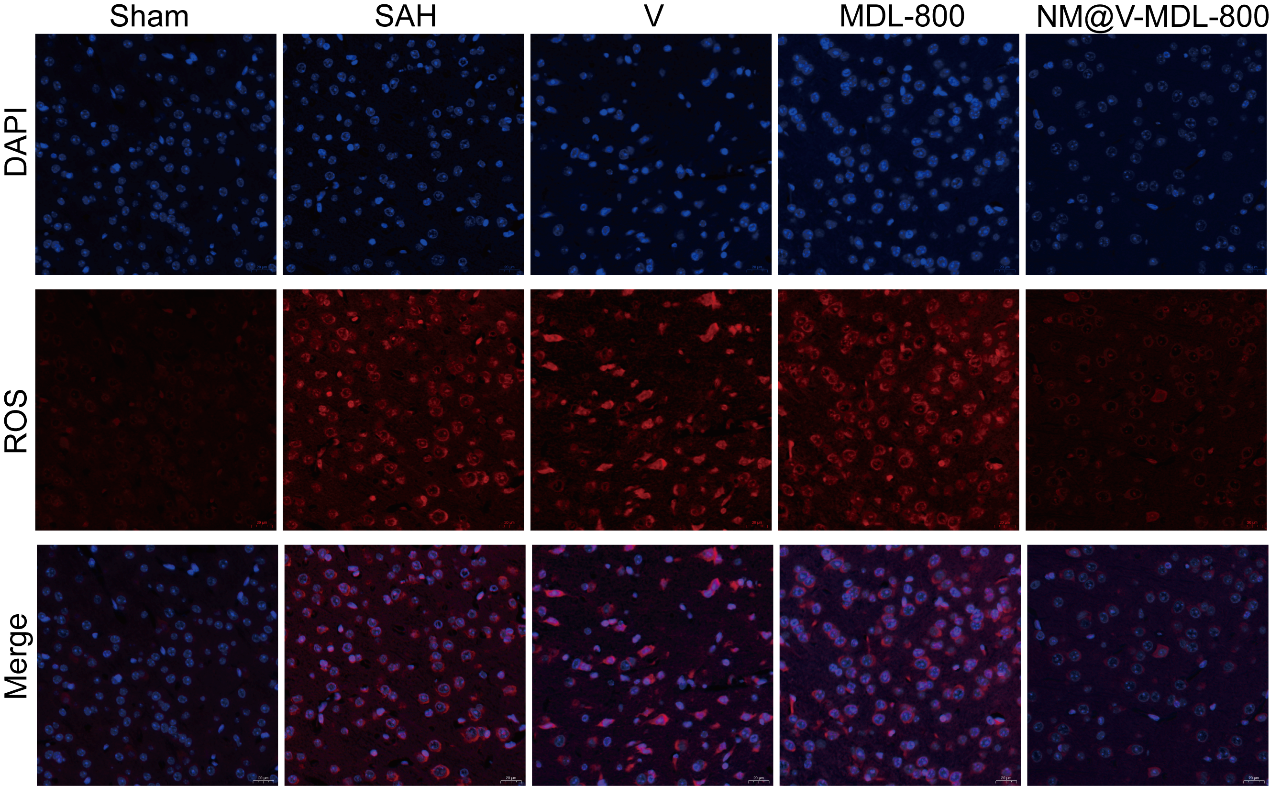


**Figure S21.** **Co-immunofluorescence staining of ROS (red) and DAPI (blue) in the SAH lesion area.**


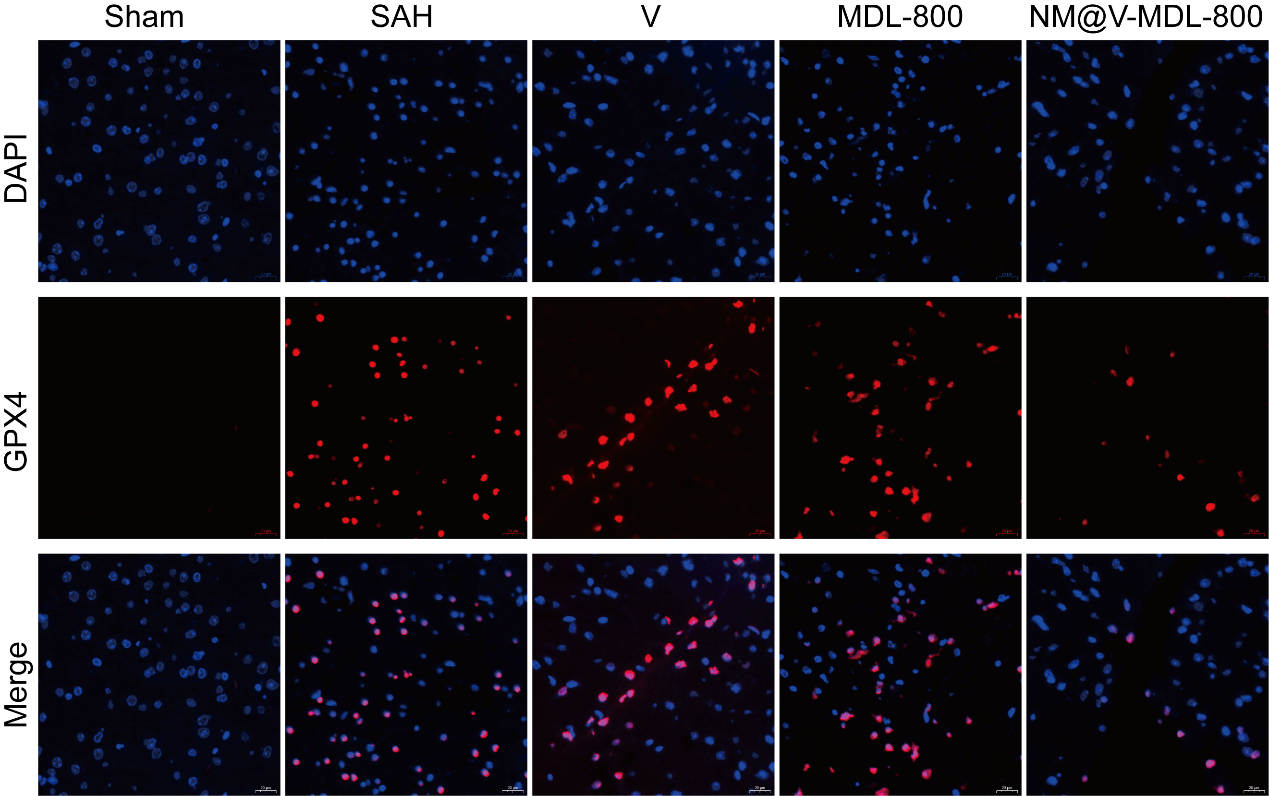


**Figure S22.** **TUNEL staining of the SAH lesion area from different groups.**


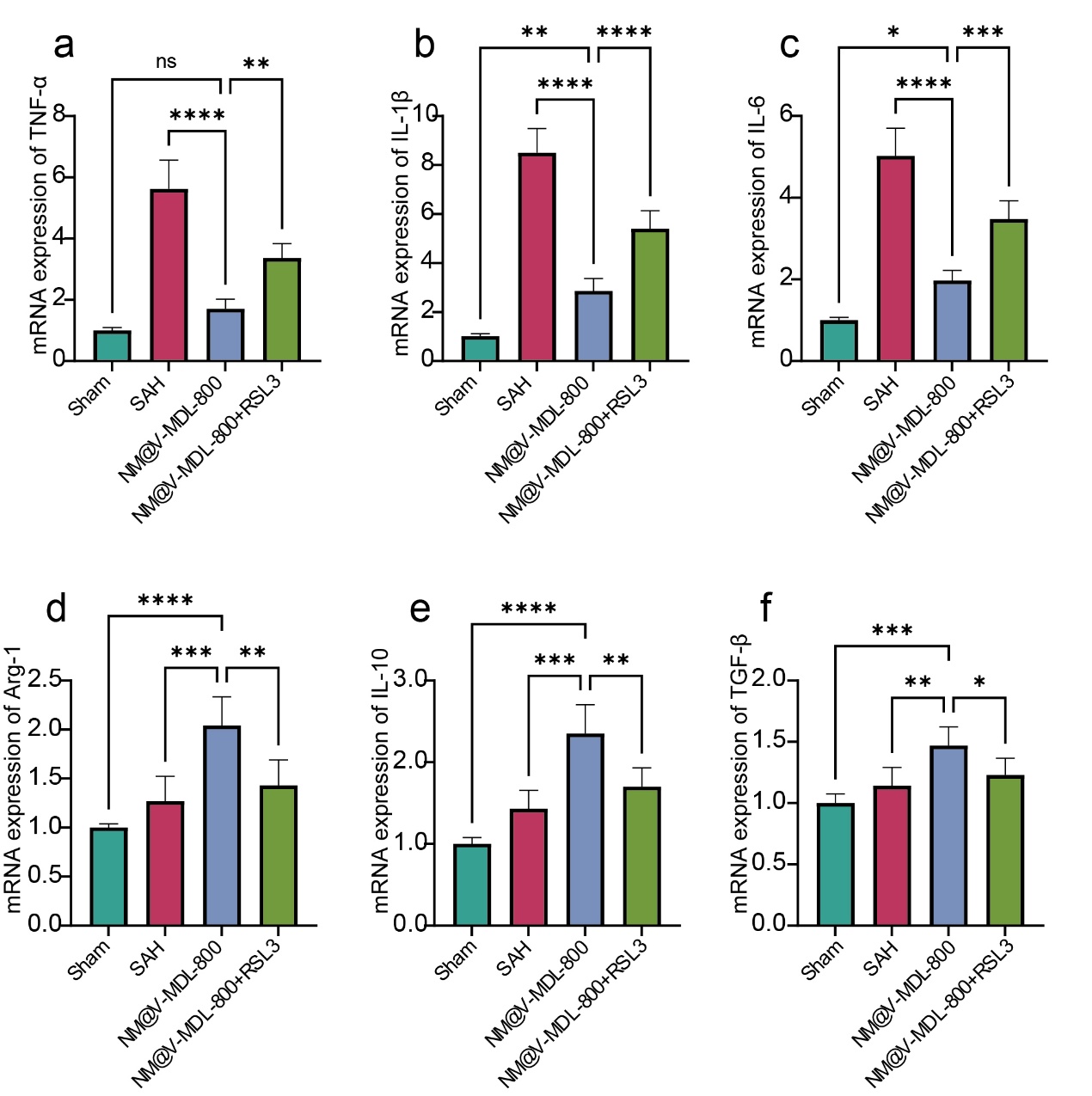


**Figure S23. The qPCR assay indicating the mRNA expression levels of TNF-a, IL-6, IL-1β, TGF-β,IL-10 and Arg-1 in vivo.**
